# Supplementary material for: Photonic-integrated quantum sensor array for microscale magnetic localisation
Source: Nat Commun. 2026 May 28;17:6185. doi: 10.1038/s41467-026-73701-0 (PMC13369943; doi:10.1038/s41467-026-73701-0)
Supplement: Supplementary file 1 — Supplementary Information [file 41467_2026_73701_MOESM1_ESM.pdf]

Supplementary Information: Photonic-integrated quantum sensor array for microscale magnetic localisation

Hao-Cheng Weng[1*], John G. Rarity[1], Krishna C. Balram[1], Joe A. Smith[2,1*]

[1]Quantum Engineering Technology Labs, H. H. Wills Physics Laboratory and Department of Electrical and Electronic Engineering, University of Bristol, Bristol BS8 1TL, United Kingdom.
[2]School of Electrical and Electronic Engineering, University of Sheffield, Sheffield S1 3JD, United Kingdom.

*Corresponding author(s). E-mail(s): haocheng.weng@bristol.ac.uk; joe.a.smith@sheffield.ac.uk;

A Supplemental result on nanodiamond positioning 2

B Supplemental result on sensor spin resonance profiles 3

C NV ensemble sensor simulations 4

D Magnetic needle tip characterisation 5

E Experimental magnetic localisation dataset 6

F Supplemental results on dynamical tracking of moving magnetic object 7

G Single-NV sensor results 8

H Microrobot simulations 9

I Scalability and operability discussion for magnetic tracking of microrobots 11

J Time-multiplexed readout of a two-dimensional NV sensor array 12

A Supplemental result on nanodiamond positioning

To demonstrate the repeatability of our nanodiamond positioning technique (see the method section in the main text), the deposition results on a different copy of the photonic chip are shown in Fig. S1. Over an array of 80 nanodiamond sites, 78 positions (a yield of 97.5%) are identified with nanodiamonds successfully positioned through the scattered light (similar to Fig. 1d in the main text).

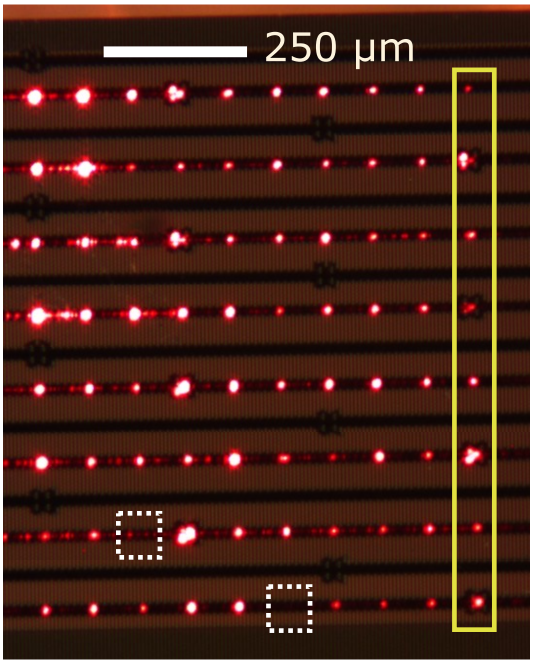

Fig. S1 Nanodiamond positioning over an array of 80 sites. Using the same deposition technique (as detailed in the method sections in the main text), 78 over 80 sites are identified with nanodiamonds successfully combined. This is visualised through the scattered light, similar to Fig. 1d in the main text.

B Supplemental result on sensor spin resonance profiles

To demonstrate the repeatability and uniformity of the NV ensemble sensors used in the experiments, we show the CW-ODMR results measured on the first column of the NV array in Fig. S1. We observed a similarly $\approx 5\%$ resonance contrast and a resonance linewidth of around ≈ 35 MHz across eight NV-ensemble sensors. Note that the CW-ODMR spectrum here is slightly affected by microwave heating.

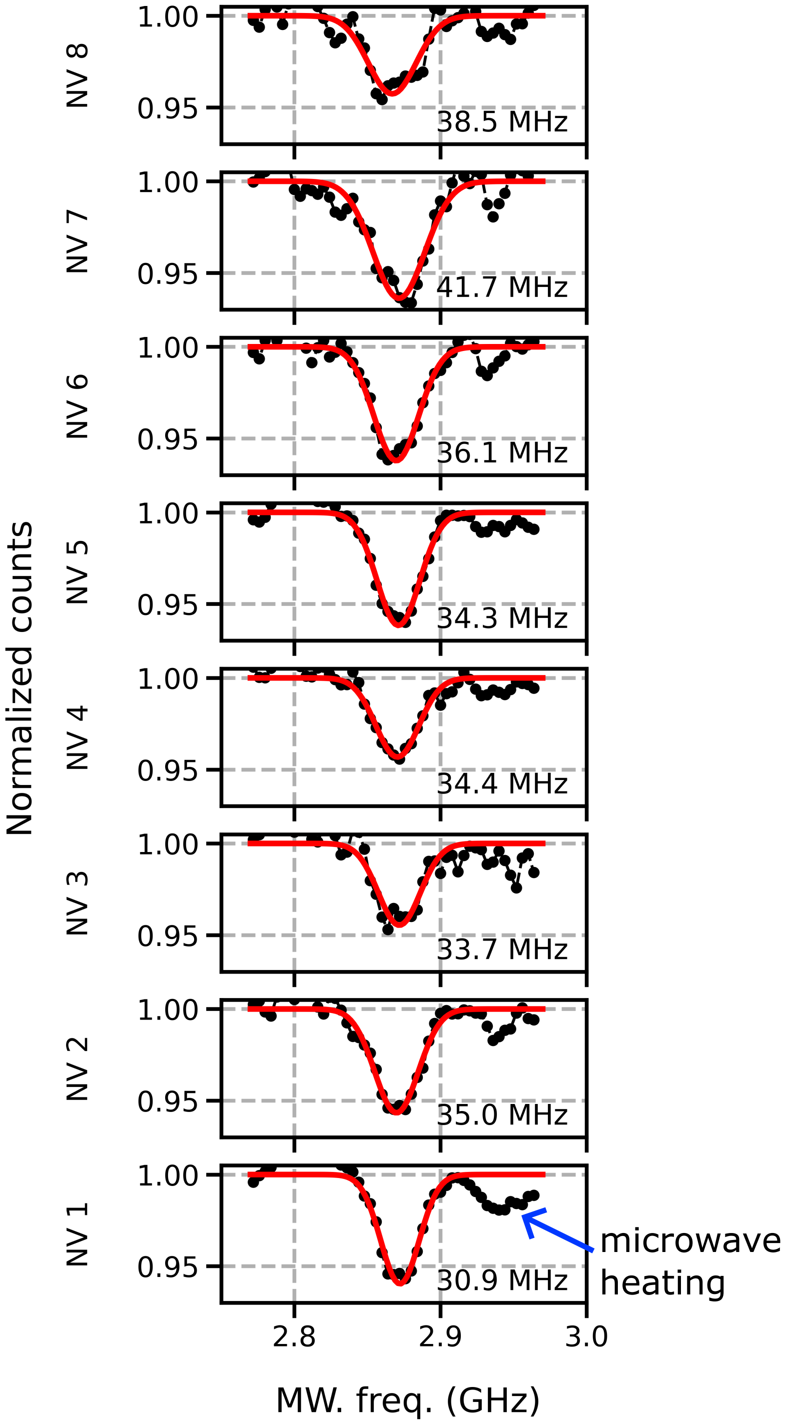

Fig. S2 Supplementary results on sensor repeatability. CW-ODMR measurements are done on the right most column (enclosed by the yellow box) in Fig. S1 in zero field. Similar resonance contrast around 5% and near uniform resonance linewidth (as labelled) are observed across the eight sensors.

C NV ensemble sensor simulations

In this section, we show simulations of NV ensemble sensors following the model given in Methods of the main text. Taking the average value of $E = 5.6$ MHz, $C = 3.5\%$, and $\delta\nu = 14.7$ MHz, we show how the Zeeman splitting (Fig. S3a), the CW-ODMR contrast (Fig. S3b), the resonance linewidth σ (Fig. S3c), and the CW-ODMR sensitivity (Fig. S3d) scale with the magnetic field strength. The Zeeman splitting is in the linear regime for $|B| \geq 0.2$ mT (with a slope of 26.9 MHz/mT), while the contrast drops below 1% for $|B| \geq 2.2$ mT. From this, we conclude the NV ensemble sensors operate with the best sensitivity in the low field regime 0.2 mT $\leq |B| \leq$ 2.2 mT.

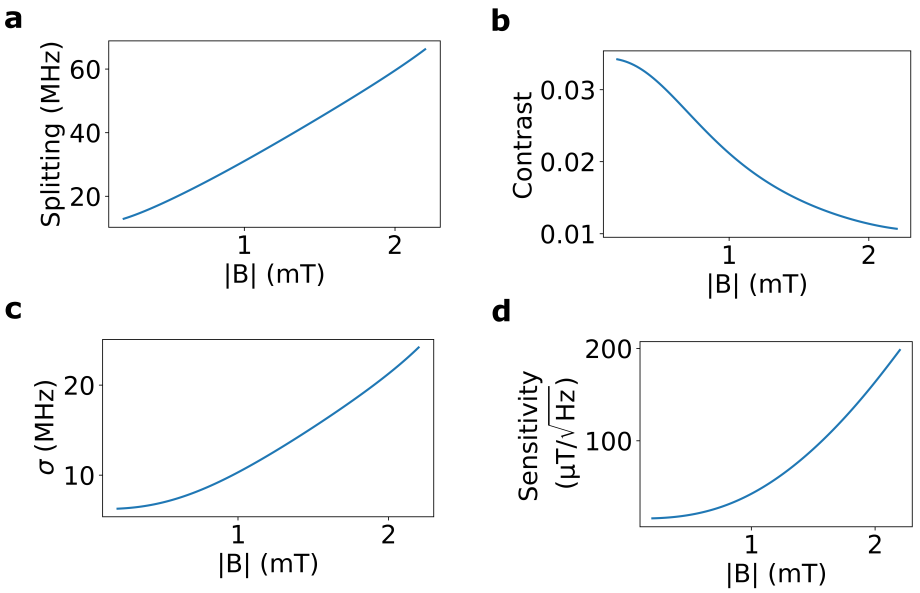

Fig. S3 Simulations of NV ensemble sensors. The **a** Zeeman splitting, **b** CW-ODMR contrast, **c** resonance linewidth σ, and **d** CW-ODMR sensitivity are plotted against the magnetic field strength under experimental parameters ($E = 5.6$ MHz, $C = 3.5\%$, and $\delta\nu = 14.7$ MHz).

D Magnetic needle tip characterisation

In this section, we provide more details for the magnetised needle tip used in the magnetic localisation experiment. A needle with a tip size of about 30 µm (characterised under optical microscope, Fig. S4a) is used to create the gradient field needed for magnetic localisation. The needle tip is first magnetised by a strong neodymium magnet for one day. In the setup, we place the needle tip around 250 µm above the chip (Fig. S4b), for safe operation, and to generate a gradient field of moderate strength (such that the NV ensemble sensors exhibit reasonable responses, see Fig. 2f in the main text). The tip is tilted at a 45° angle for ease of setting up and scanning with the motorised stage (not shown in the figure).

To characterise the gradient field generated by the tip, we scan the tip across the operation area while recording the Zeeman splitting of the eight sensors (Fig. S4c). This allows us to extract the X-direction gradient $\approx 4.2 \times 10^{-3}$ mT/µm and the Y-direction gradient $\approx 1.7 \times 10^{-3}$ mT/µm. The gradients are calculated at the cross positions indicated in Fig. S4c. The smallest gradient among the eight sensors is taken and averaged over different positions.

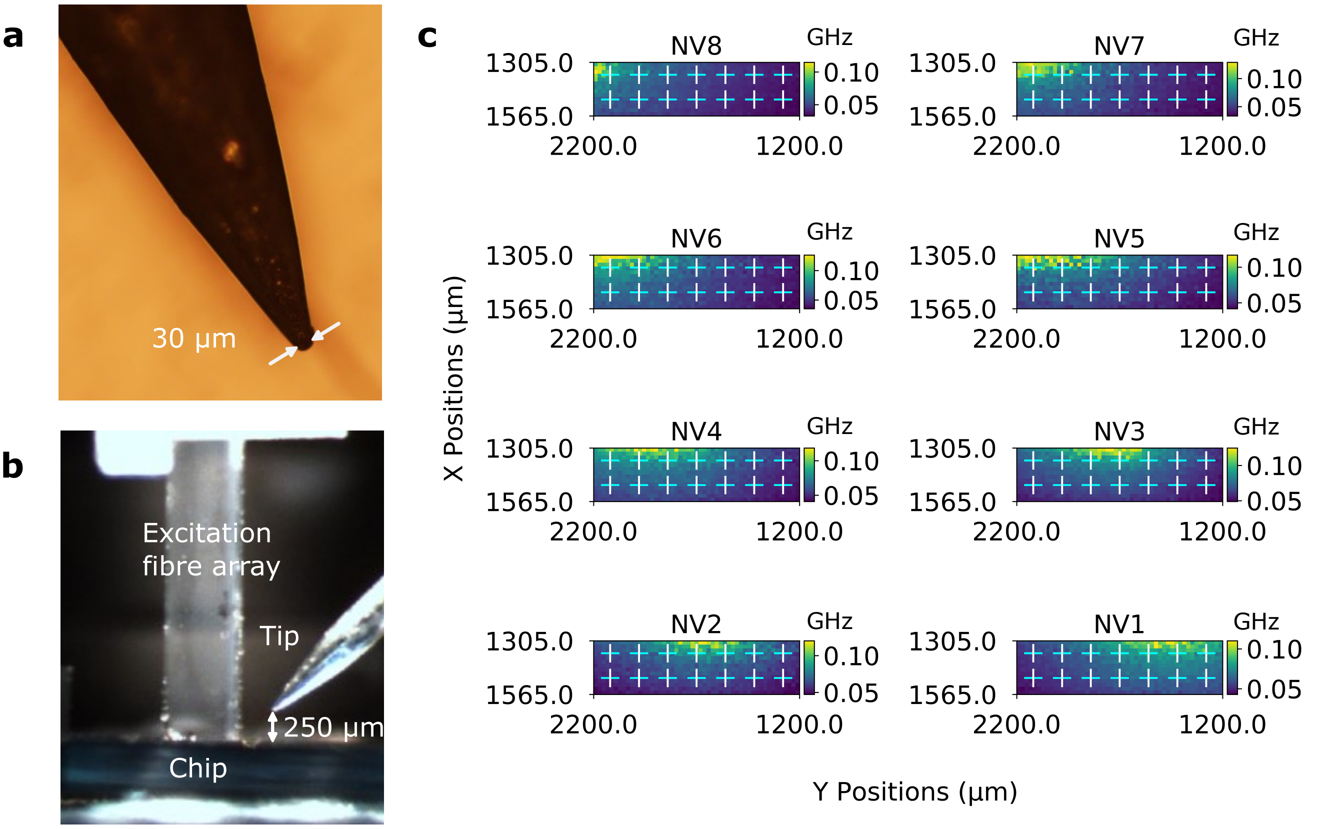

Fig. S4 Characterisation of the needle tip and field gradient. **a** Optical-microscope image of the magnetised needle tip. **b** Image of the experimental setup for magnetic localisation and dynamic tracking demonstrations. **c** The Zeeman splitting values (in GHz) recorded by the eight sensors over the operation area. The field gradients are calculated at the cross positions.

E Experimental magnetic localisation dataset

For completeness, the dataset used to train the machine learning model and contribute to the experimental results presented in Fig. 3 of the main text is shown. The needle position is sampled evenly across the operation area, with 90% for training, 7% for validation, and 3% for testing and visualisation in Fig. 3d of the main text. The dataset is visualised in Fig. S5 by the sampled needle tip position (true positions in Fig. 3d in the main text), demonstrating the train-validation-test separation.

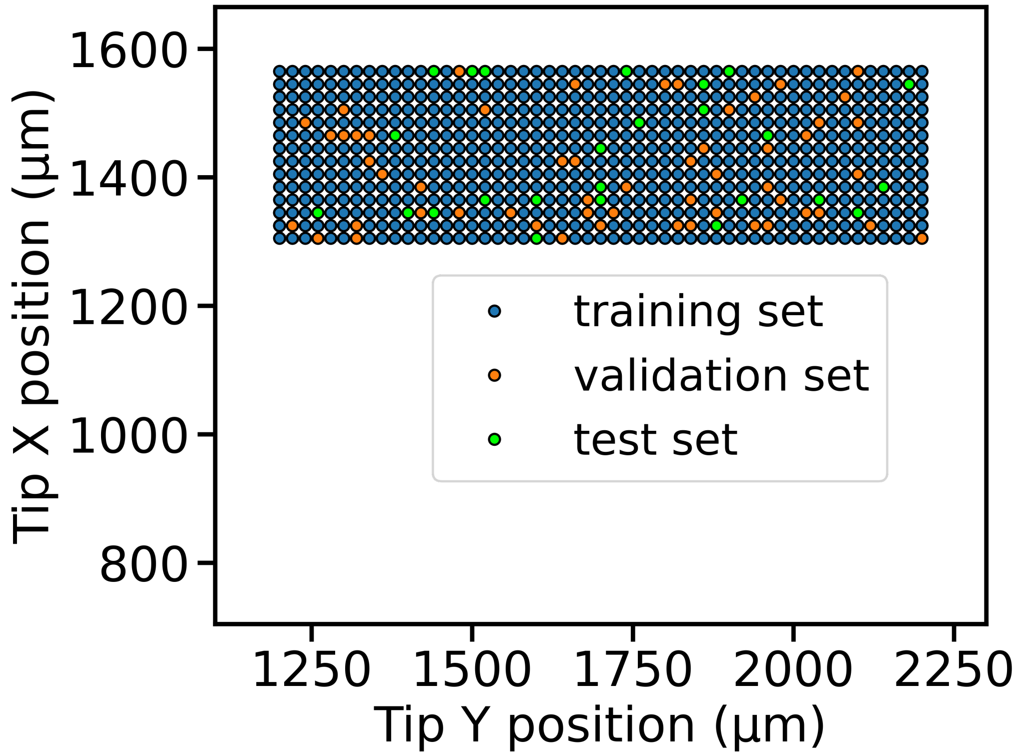

Fig. S5 Visualisation of the dataset by the sampled (true) needle tip positions. The dataset that contributes to the results in Fig. 3 of the main text is shown. Some of the positions are sampled for more than once for a better quality of the measurement. The training dataset is separated from the validation set and the test set.

F Supplemental results on dynamical tracking of moving magnetic object

We provide supplemental results the experiments in Fig. 4 of the main text. The Mean Perpendicular Distance (MPD) is calculated for different tip moving speeds and frame rates. Considering both smearing due to under sampling and the shot-noise effect, MPD increases from the lower left to the upper right of the figure as illustrated.

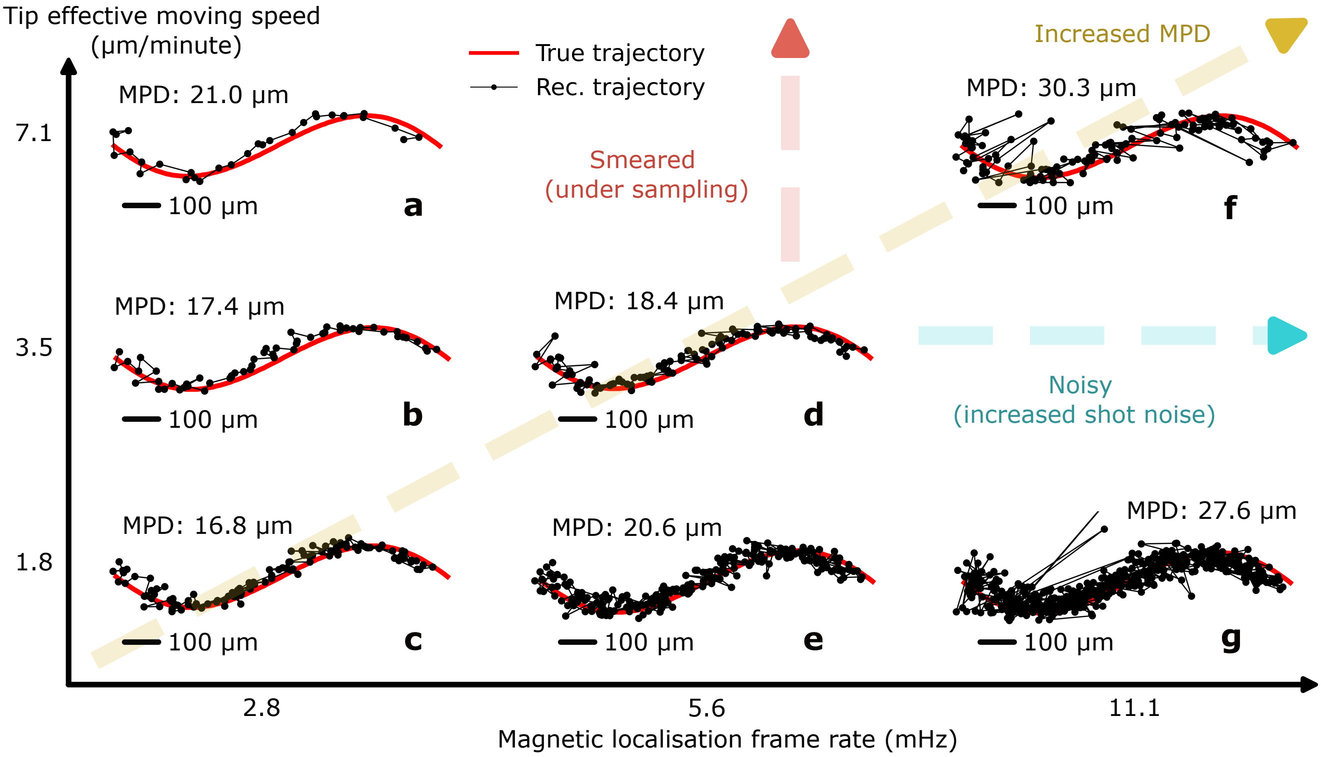

Fig. S6 Supplemental results on experimental dynamical tracking of moving magnetic object. The results presented in **c, b, a** and **c, e, g** corresponds to that presented in Fig. 4a, b, c and Fig. 4d, e, f of the main text, respectively.

G Single-NV sensor results

Our photonic platform (Fig. 1 in the main text) for multi-NV quantum sensing applies both to the NV-ensemble sensors and single-NV sensors. In this section, we show the characterisation of single-NV quantum sensors from higher-purity nanodiamonds [1], deposited on the same silicon nitride photonic chip and following the same excitation and collection scheme in Fig. 1a in the main text. With <4 mW pump power used, we observe the photoluminescence counts around 6000/s recorded from one end of the waveguide (Fig. S7a). Note that this is below the saturated emission. In the single NV regime, the fluorescence from the waveguide and fibre could contribute to noise in the system as discussed in [1], causing the extended Gaussian spot in Fig. S7a.

We perform Continuous-Wave Optically-Detected-Magnetic-Resonance (CW ODMR) measurements on the single NV. The splitting under zero field (Fig. S7b) is due to strain in the positioned nanodiamond. With an external field (not aligned to the NV centre), further splitting is observed (Fig. S7c) due to the Zeeman effect. This helps to verify that the Gaussian spot is from a single NV since the lower nitrogen content of this nanodiamond suggests a few percent NVs per nanodiamond [2] and one would see two pairs of dips if the NVs are from two nanodiamonds of different orientations. In Fig. S7d, we also perform Rabi oscillations on the NV spin to show coherent controllability of the spin for pulsed magnetometry protocols.

Based on these results, we can calculate the single-NV CW ODMR sensitivity [3] following $\eta_{\mathrm{CW-ODMR}} = \frac{4}{3\sqrt{3}} \frac{h}{g_e \mu_{\mathrm{B}}} \frac{\Delta\nu}{C_{\mathrm{cw}} \sqrt{R}}$, where h is the Planck constant, g_e the electron g-factor, μ_{B} is the Bohr magneton, $\Delta\nu$ is the resonance linewidth (FWHM), C_{cw} is the CW-ODMR contrast, and R is the photon-detection count rate. We estimate the single-NV sensitivity to be $\approx 25\ \mu\mathrm{T}/\sqrt{\mathrm{Hz}}$. This number is used in the simulations of the microrobot tracking. Note that the sensitivity can be improved by optimising the optical pump power, adopting longer-coherence-time single NVs in nanodiamonds [4], and using other magnetometry protocols such as Ramsey interferometry [3].

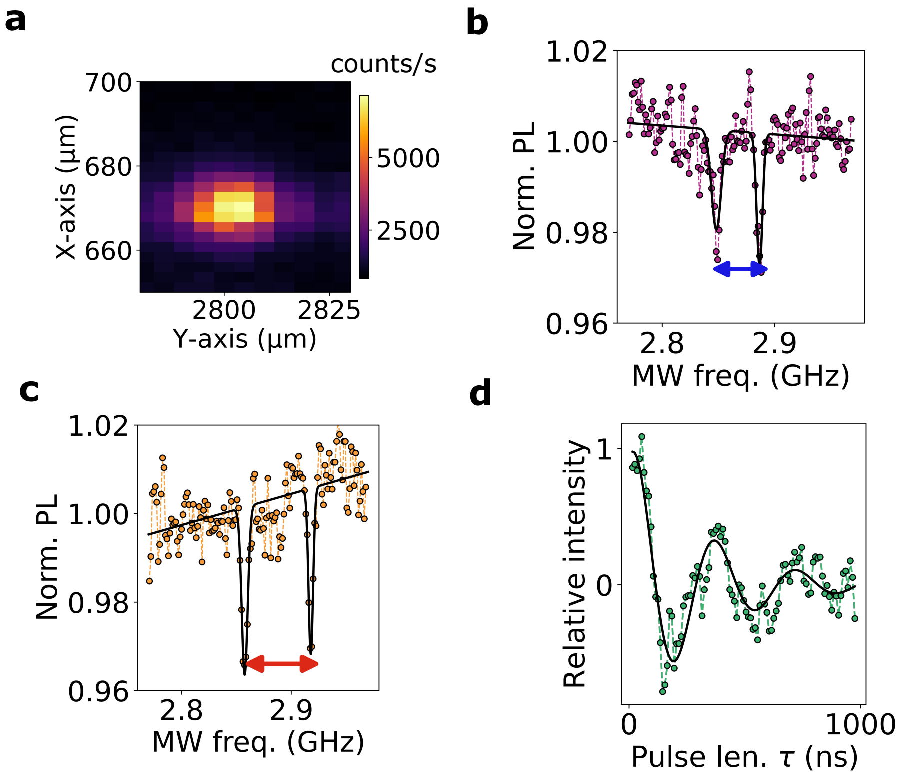

Fig. S7 Single-NV quantum sensor characterisation. a Photoluminescence counts of a single NV collected from a waveguide when the excitation fibre is scanned across the device surface plane. **b** CW ODMR measurement of the single NV under zero field. The splitting (blue arrow) is caused by strain in the nanodiamond. The normalised photoluminescence count is plotted against the microwave frequency. **c** CW ODMR measurement of the single NV under external magnetic field, presenting a larger splitting (red arrow). Here the field is not aligned with the NV axis, resulting in collective shifting of the ±1 electron spin resonances under strain. The normalised photoluminescence count is plotted against the microwave frequency. **d** Rabi oscillations of the NV centre at 2.86 MHz. The relative photoluminescence intensity is plotted against the length of the resonant microwave pulse τ.

H Microrobot simulations

We simulate the magnetic field profile generated by a magnetic hydrogel robot [5] in COMSOL Multiphysics. We consider a 100 µm diameter sphere made of isotropic ferrite, magnetised along the z-direction (when the rotation angles are set to $\theta = 0$ and $\phi = 0$). In Fig. S8, we show the magnetic field generated by this microrobot at a plane 200 µm below it.

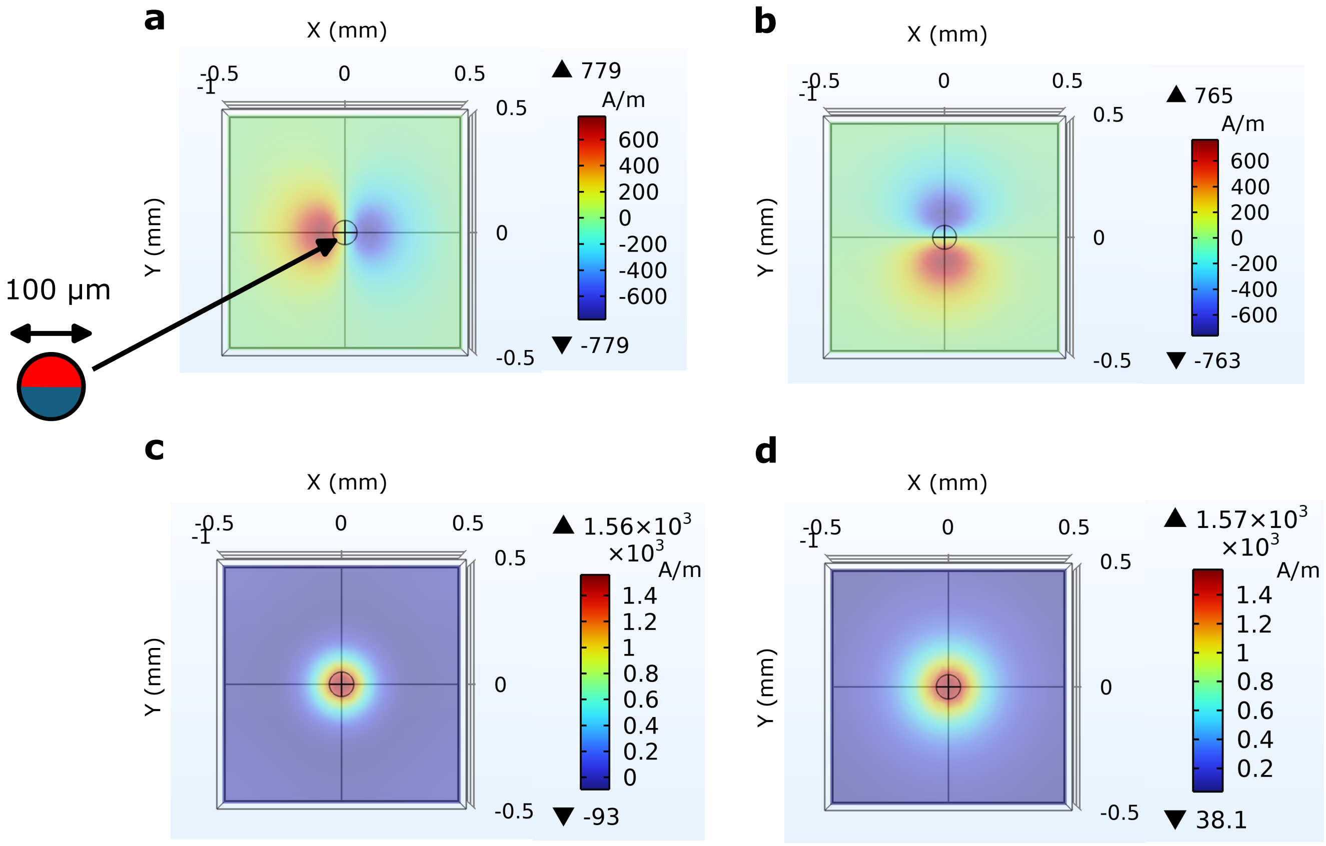

Fig. S8 Simulation of the magnetic field profile from a 100-µm spherical ferrite microrobot. The magnetic field in the x direction **a**, the magnetic field in the y direction **b**, the magnetic field in the z direction **c**, and the magnetic field magnitude (absolute value) **d**. The sphere is magnetised in the z direction and the field (in units of A/m) is recorded at a plane 200 µm below the microrobot.

For magnetic localisation with NV ensemble sensors (Fig. 5b and Fig. 5c in the main text), the magnetic field magnitude is sampled with microrobot positions within the -1400 µm $\leq$ x $\leq$ 0 µm and -700 µm $\leq$ y $\leq$ 700 µm area for 5041 training data points. The eight sensors are located at x=0 µm, y= (3-n)$\times$ 127 µm for n=0-7, and vertically 250 µm below the microrobot. See Fig. 5b in the main text for visualisation. In this simulation, the microrobot orientation remains fixed (at $\theta = 0$ and $\phi = 0$). To consider the effect of sensor sensitivity, we take the field-strength-dependent sensitivity (measured in Fig. 2f in the main text) and apply Gaussian random noise at the sensitivity level to the exact value (i.e. $B_{\text{noisy}} = B_{\text{exact}}$ + Gaussian_rand_noise[$t = 360$, $\eta_{\text{CW-ODMR}}$]). The labeled data is split such that 95% is used for training and 5% is used for validation. To visualise the result, a new set of data points are generated over the same area but with the sampling positions shifted slightly. The estimation error is plotted as a heatmap for visualisation in Fig. 5b in the main text. We use a similar machine learning model architecture, with three Conv1D layers of 128, 256, 512 dimensions, each followed by ReLU activation function. Four Dense layers are then used (of 512, 128, 64, 2 dimensions) after flattening. An Adam optimiser is used with learning rate 0.001 and the model is trained for 300 epochs with a batch size of 128. Note that the error map is averaged over 20 independent training runs of the model to reduce stochastic effects.

For position and orientation tracking with single NVs (Fig. 5d and Fig. 5e in the main text), the magnetic vector field is sampled with microrobot positions within the -750 µm $\leq$ x $\leq$ -50 µm and -350 µm $\leq$ y $\leq$ 350 µm area. The eight sensors are located at x=0 µm, y= (3-n)$\times$ 127 µm for n=0-7, and vertically 150 µm below the microrobot. We sample 1296 positions in the xy plane, along with 91 angles from θ=0 to 180° ($\phi = 0$ remains fixed). This gives us a total of 1296×91 data points for positions and orientations. We consider single NV sensors

of random orientations and $|\vec{B \cdot \hat{a}}|$ is calculated for each sensor where $\hat{a}$ is the NV orientation. We then similarly add Gaussian random noise to the exact values to consider the single-NV sensitivity (as discussed in Section C). A similar training model is used except now the model is trained to learn not only the microrobot position but also the θ angle. With a larger dataset in use (50% of the dataset is used for training and 50% for validation), we train the model only for 50 epochs. To showcase the microrobot position and orientation tracking, we sample a trajectory of the microrobot movement, along with controlled rotations. This is presented in Fig. 5d and Fig. 5e in the main text. Note these data points are not included in the training set. We also verify the microrobot position and orientation tracking for three randomly sampled sets of NV sensor directions, with minimal variations observed. To experimentally realise magnetic localisation with single-NVs, it is necessary to characterise the single-NV axes, for example, following [6]. In our current setup, the amount of pump laser power required to enter the saturation regime (estimated 10 to 100 times) prevents us from implementing parallel single NV sites.

I Scalability and operability discussion for magnetic tracking of microrobots

As proposed in Fig. 5d and Fig.5e of the main text, single-NV sensors can enable position and angular tracking simultaneously. Here, we discuss how an array of eight single-NV sensors can be created on the chip-based platform.

As shown in [7], for the same nanodiamond type we take the probability of obtaining at least one NV at a successfully positioned site to be $P(\geq 1) \approx 0.44$. Modelling the NV count per site as Poisson with mean λ gives $\lambda \approx 0.58$, and therefore $P(0) = e^{-\lambda} \approx 0.56$, $P(1) = \lambda e^{-\lambda} \approx 0.33$, and $P(\geq 2) = 1 - P(0) - P(1) \approx 0.11$. Combining this with the nanodiamond positioning yield of 97.5% (Fig. S1), the per-site probabilities are approximately $p_1 \approx 0.975 \times 0.33 \approx 0.32$ (single NV), $p_0 \approx (1-0.975)+0.975\times 0.56 \approx 0.57$ (empty/no NV), and $p_{\geq 2} \approx 0.975\times 0.11 \approx 0.11$ (multiple NVs).

In the following estimate we treat multi-NV sites as not meeting the single-NV condition and allow repeat deposition only when a site is empty/no-NV. If we allow up to x independent deposition attempts, the probability that a given site ends up as a single-NV site is $p_{\mathrm{site}}(x) = p_1 \sum_{k=0}^{x-1} p_0^k = p_1(1-p_0^x)/(1-p_0)$, and the probability that all eight sites form a single-NV array is $p_{\mathrm{array}}(x) = p_{\mathrm{site}}(x)^8$. For example, with $x = 4$ we obtain $p_{\mathrm{site}} \approx 0.67$ and thus $p_{\mathrm{array}} \approx 0.04$, corresponding to ~ 1 such array expected when screening ~ 25–30 arrays.

We note that this estimate is conservative, since sites with more than one NV may still be usable in practice by applying a bias magnetic field to spectrally separate NV orientations and selecting a subset of resonances for sensing, as is commonly done in bulk-diamond NV magnetometry.

In the main text, it is shown that the microrobot tracking frame rate can be improved significantly by improving the sensor sensitivity to around 1 nT/$\sqrt{\mathrm{Hz}}$ [8, 9]. This presents a 10^5 sensitivity improvement over the 100 µT/$\sqrt{\mathrm{Hz}}$ shown by our current NV sensors. To see how 1 nT/$\sqrt{\mathrm{Hz}}$ sensitivity can be explicitly achieved in our platform, we can consider the integration of chemical vapour deposition (CVD)-grown diamond membranes (300 nm thick) on photonic waveguides. Commercial CVD diamond (DNV-B14, Element 6) has a high concentration of NVs (4.5 ppm of NVs) and T_2^* coherence time of 0.5 µs at room temperature [9]. This coherence results in 50 times narrower linewidth $\Delta\nu = 1/(\pi T_2^*)$ than measured in this work. The increased NV concentration of this material implies 2.5×10^5 NVs at a positioned site of 4 µm × 0.4 µm × 0.3 µm (300 nm thick). At saturation, this contributes $\approx$ 9 GHz count rate in our system (estimated by the saturated rate of a single NV in [7] and the improved collection efficiency presented in Fig. 1g of the main text). This presents an improved increased optical flux of 5×10^4 compared to the measured signal in our work. Following $\eta_{\mathrm{CW-ODMR}} \propto \frac{\Delta\nu}{\sqrt{R}}$,, with adopting improved NV linewidths and NV density, the CW-ODMR sensitivity can thus be improved by ≈ 1000, and again by over ten times by considering pulsed ODMR sequences [8].

We further show that the improved sensitivity can benefit both the frame rate and the operation range. Following Eq. 2 of the main text ($R_{x,y} \approx S_{x,y}/(\sqrt{t}\ m_{x,y})$), the 10^5 smaller sensitivity can support a 10^4 shorter integration time and the detection of a 10^3 weaker field gradient for the same resolution. The tracking frame rate, inversely proportional to the integration time, can thus be improved by four orders of magnitude. The operation range is related to the magnetic field gradient, as shown in Fig. 5c of the main text. Following Biot-Savart law, where $B \propto 1/r^2$ and r is the distance from the sensor to the magnetic object, the magnetic gradient $dB/dr \propto 1/r^3$ means that the ability to detect a 10^3 weaker field allows ten times the operation range. Note that this applies to both the X and Y ranges in Fig. 5b. In conclusion, we show that by improving the sensor sensitivity to 1 nT/$\sqrt{\mathrm{Hz}}$ level, the microrobot tracking frame rate can be boosted by 10^4 times, at the same time, with the operation range extended by ten times.

J Time-multiplexed readout of a two-dimensional NV sensor array

In this section, we elaborate in detail how the photonic scheme presented in Fig. 1 of the main text can be scaled to a two-dimensional array controlled by an eight by eight matrix fibre array (as shown in Fig. S9a). To realise parallel operation of sensors and distinct readout, we utilise time-multiplexed readout for NV sensors coupled to the same waveguide (similar to [10]). The working principle is summarised by Fig. S9b, considering Ramsey interferometry type measurements for magnetic sensing [3]. All sensors are first polarised by a green laser pulse. With a common microwave drive (similar to the PCB antenna used in Fig. 1b of the main text), the spin states are rotated into superposition states and projected for readout. Notably, all sensors experience the same interrogation time. Lastly, the spin state readout for NVs coupled to the same waveguide is multiplexed. The number of spins on the same waveguide (that can be multiplex readout) is only limited by the spin coherence time, and up to 108 spins have been demonstrated for single NVs in room temperature [10]. For the eight by eight NV array, we visualise the pump pulses separately applied to each column of the fibre array. As a result, the NV centres on the same waveguide are sequentially readout, as shown in the PL signal. With eight spatial channels (waveguides) and eight readout time instances, the 64 sensor's information (over the same interrogation time) can be distinctly and efficiently extracted.

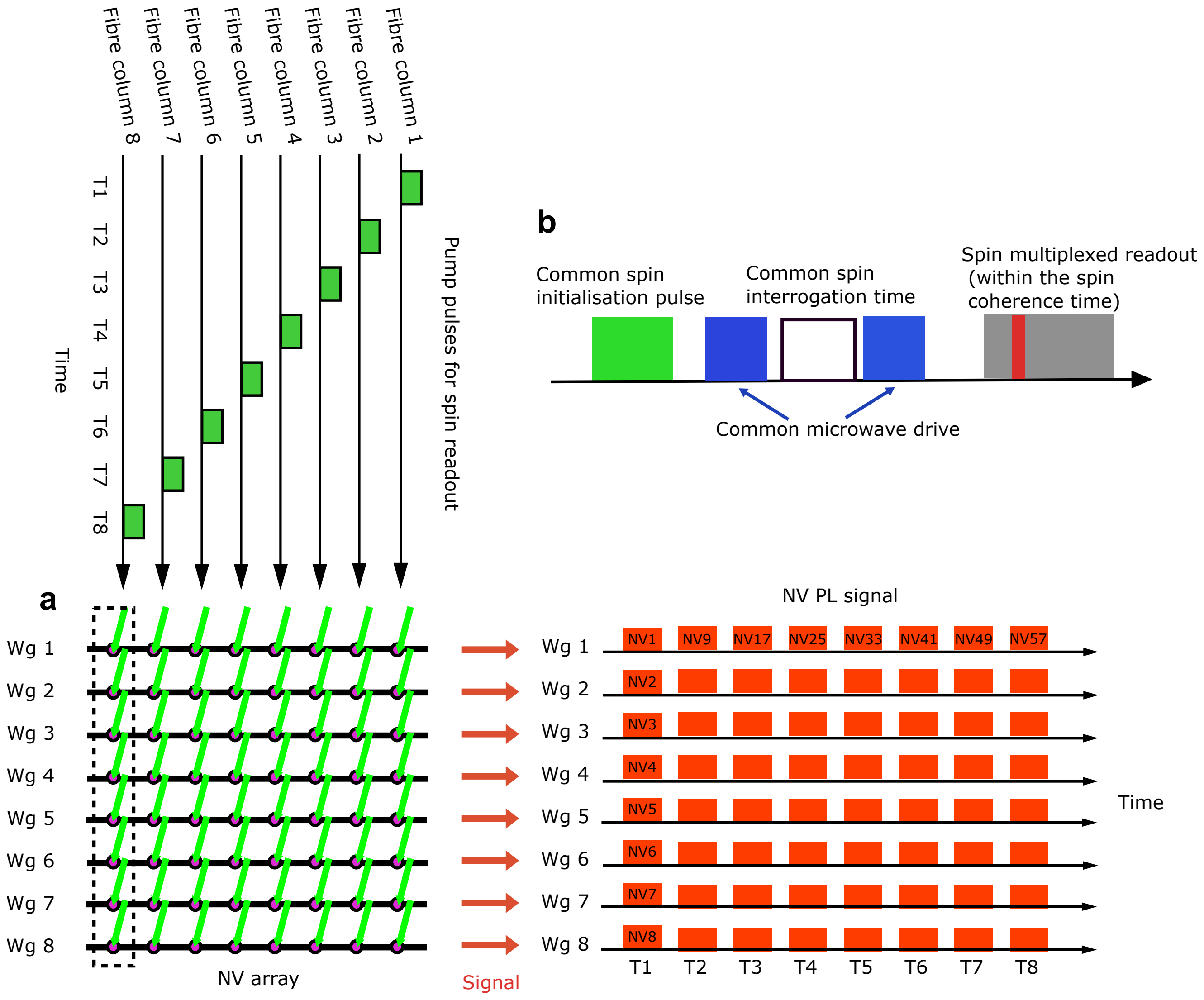

Fig. S9 Time multiplexed readout of a two-dimensional sensor array. **a** An eight by eight NV array connected by eight waveguide (Wg 1- wg 8) channels and optically excited by matrix fibre arrays. The pump pulse for each column of the matrix fibre are shown for sequential spin readout. The PL signal from the 64 sensors are distinguished between eight physical waveguide channels and eight time instances (T1-T8). **b** The working principle of multi-NV quantum sensing with time-multiplexed readout.

References

[1] Weng, H.-C., Monroy-Ruz, J., Matthews, J.C.F., Rarity, J.G., Balram, K.C., Smith, J.A.: Heterogeneous integration of solid-state quantum systems with a foundry photonics platform. ACS Photonics **10**(9), 3302–3309 (2023)

[2] Knowles, H.S., Kara, D.M., Atatüre, M.: Observing bulk diamond spin coherence in high-purity nanodiamonds. Nature materials **13**(1), 21–25 (2014)

[3] Barry, J.F., Schloss, J.M., Bauch, E., Turner, M.J., Hart, C.A., Pham, L.M., Walsworth, R.L.: Sensitivity optimization for nv-diamond magnetometry. Reviews of Modern Physics **92**(1), 015004 (2020)

[4] March, J.E., Wood, B.D., Stephen, C.J., Fervenza, L.D., Breeze, B.G., Mandal, S., Edmonds, A.M., Twitchen, D.J., Markham, M.L., Williams, O.A., *et al.*: Long spin coherence and relaxation times in nanodiamonds milled from polycrystalline 12 c diamond. Physical Review Applied **20**(4), 044045 (2023)

[5] Jiang, F., Zheng, Q., Zhao, Q., Qi, Z., Wu, D., Li, W., Wu, X., Han, C.: Magnetic propelled hydrogel microrobots for actively enhancing the efficiency of lycorine hydrochloride to suppress colorectal cancer. Frontiers in Bioengineering and Biotechnology **12**, 1361617 (2024)

[6] Fukushige, K., Kawaguchi, H., Shimazaki, K., Tashima, T., Takashima, H., Takeuchi, S.: Identification of the orientation of a single nv center in a nanodiamond using a three-dimensionally controlled magnetic field. Applied Physics Letters **116**(26) (2020)

[7] Weng, H.-C., Monroy-Ruz, J., Matthews, J.C., Rarity, J.G., Balram, K.C., Smith, J.A.: Heterogeneous integration of solid-state quantum systems with a foundry photonics platform. ACS photonics **10**(9), 3302–3309 (2023)

[8] Zhang, Y., Li, Z., Feng, Y., Guo, H., Wen, H., Tang, J., Liu, J.: High-sensitivity dc magnetic field detection with ensemble nv centers by pulsed quantum filtering technology. Optics Express **28**(11), 16191–16201 (2020)

[9] Guo, Y., Coccia, G., Kavatamane, V.K., Giakoumaki, A.N., Vetlugin, A.N., Ramponi, R., Soci, C., Barclay, P.E., Hadden, J.P., Bennett, A.J., *et al.*: Enhanced quantum magnetometry with a femtosecond laser-written integrated photonic diamond chip. Nano Letters **25**(20), 8096–8102 (2025)

[10] Cambria, M., Chand, S., Reiter, C.M., Kolkowitz, S.: Scalable parallel measurement of individual nitrogen-vacancy centers. Physical Review X **15**(3), 031015 (2025)
