# Supplementary material for: Photonic-integrated quantum sensor array for microscale magnetic localisation
Source: Nat Commun. 2026 May 28;17:6185. doi: 10.1038/s41467-026-73701-0 (PMC13369943; doi:10.1038/s41467-026-73701-0)
Supplement: Supplementary file 2 — Transparent Peer Review file [file 41467_2026_73701_MOESM2_ESM.pdf]

nature portfolio

Peer Review File

Photonic-integrated quantum sensor array for microscale magnetic localisation

Corresponding Author: Dr Joe Smith

This file contains all reviewer reports in order by version, followed by all author rebuttals in order by version.

Version 0:

Reviewer comments:

Reviewer #1

(Remarks to the Author)
In this manuscript, H.-C. Weng et al. demonstrate the simultaneous use of eight separate NV nanodiamond ensembles on a silicon-nitride photonic circuit. These sensors are used to localize a ~ 30 um-sized magnetic tip without direct optical access, using machine learning methods. The localizing ability improves with the number of sensors used. The authors suggest that this platform is appropriate for tracking the position and rotation of magnetic objects in biological systems on the millihertz timescale, but only if the NV nanodiamond ensembles are replaced with single NV center arrays or possibly single-crystal NV ensemble arrays. While I think this parallelized approach is a step towards remote tracking of optically inaccessible magnetic objects, I am not convinced this manuscript merits publication in Nature Communications. Below, I have outlined my concerns.

Major concerns:
1. This work does not show a substantial technical advance and there is no fundamentally new science gained. This work is a follow-up to a previous work (Ref. 15, by the same authors) and the basic experimental principles and techniques are not significantly different; A MW antenna, improved optical coupling efficiency, and parallel sensor operation (similar to Refs. 7-14) are the main additions. The ~50 uT/sqrt(Hz) sensitivity and ~1 mT dynamic range are not remarkable compared to existing literature. Additionally, the use of machine learning for magnetic tracking with NV centers is not a new idea (see Meneses et al. 2025 doi.org/10.48550/arXiv.2502.14683).
2. I do not believe the experiments demonstrated support the claims that this platform can be used for dynamic position and rotation tracking of magnetic objects with an array of single NV sensors. The magnitude-based NV ensembles cannot track rotations, and I do not see how nanodiamond drop casting will repeatably result in arrays of single NV centers. Moreover, the random orientation of each NV center would need to be calibrated before use. The author should demonstrate parallelized sensing with single-NV arrays to suggest the feasibility of position and rotation tracking.
3. Machine learning methods require massive amounts of training data that i) is highly sensitive to the magnetic field of the object and ii) includes knowledge of the object’s position during measurement (which is already the point of the measurement). How do the authors suggest conducting tracking experiments, if the position, orientation, and type of magnetic object is unknown from the start?
4. With parallelized multi-NV measurements, theoretical and practical scalability should be addressed. The authors claim this platform is scalable, but no discussion about scalability is given. What potential bottlenecks do the authors foresee (low yield of single-NV nanodiamonds, size resolution for the excitation fiber array, etc.)?

Minor concerns:
1. The authors should include a comparison to the works of S. Chen et al. 2021 (doi.org/10.1073/pnas.2118876119) and N. Mathes et al. 2024 (doi.org/10.1039/D3NA00684K) that images micro/nano-sized magnetic particles inside a biological sample with NV centers.
2. The authors refer to eight NV sensors in the text. However, each sensor is not an NV center but an ensemble of many nanodiamonds containing multiple NV centers. The authors should clear up this ambiguity throughout the abstract and text.
3. Just how far below optical saturation do the NV ensembles operate? Are saturation curves available?
4. Why does adding more sensors improve the reconstruction linearly in figure 3f? A naïve assumption would be that it improves proportional to the square root of the sensors.
5. In figure 4, the tip is not moving at the indicated speed, but instead takes discrete steps every X seconds. The authors should mention that measurements without smearing present an idealized version of motion tracking and should change “tip

moving speed" to "effective tip speed".
6. In figure 5b, the authors should consider a contour line to show the < 50 um region, or use multiple contours, rather than a box to denote their "good operating area".
7. For completeness, the authors should include a supplemental figure with the ~ 750 tip positions used in figure 3d-f for reference. Additionally, they should show the separation of that data into the training/validation subsets that produced figure 3h.

Typos/mistakes:
1. "TE-mode" should be written out fully when first introduced.
2. Refs. 10 and 13 should be updated, as both are now published.
3. The color bar labels are missing in supplementary figures 2, 3, and 4.

Reviewer #2

(Remarks to the Author)
First of all, I would like to thank the authors for the manuscript. However, the comments below have to be addressed by the authors.
1. How much is the green pump rejection in the experiment? It would be helpful to quantify this.
2. Is there a reason to place the nanodiamond area in the middle of the Silicon-nitride waveguides compared to one of the ends?
3. For the diamond deposition, do you remove the PMMA mask after deposition? In the text, it is mentioned that "residual nanodiamonds on the PMMA mask are then removed by acetone", does this mean acetone could remove the wanted deposited nanodiamonds? Also, what is the repeatability of this technique?
4. It would be beneficial for the reader to explain how Fig 1g is calculated. Is it measured or simulated data? Does it consider the coupling efficiency of the NV fluorescence to the waveguides as well or just the waveguides to the collection fibers? Please elaborate in the manuscript. Waveguide-to-chip coupling is defined as 85%, however in Fig. 1g the coupling efficiency is around 25% maximum. In addition, is waveguide-to-chip coupling the same as fiber to on-chip waveguide coupling?
5. The authors mentioned "the physical size of the sensor (and thus the ultimate spatial resolution for quantum sensing) is defined directly by the deposition area (which is 4 μm by 500 nm)." Is it not defined by the fiber array pitch (127 um)? The sensor can't resolve such small resolution that is claimed in the manuscript.
6. The statement "the average slope is 22 ± 3 MHz/mT, which is about half the maximum splitting (2 × 28 MHz/mT) due to the averaged response of isotropic NV orientations" is not very clear. Is there a prior experiment in the literature with such performance? Does it make sense that the average response of the nanodiamonds changes the effective gyromagnetic ratio this much?
7. It is not clear why the sensitivity doubled when magnetic field changes from 1mT to 1.5mT. This sounds like a very big change.
8. It would be helpful for the reader to elaborate more on why the estimation error of the magnetic localisation experiment reduces linearly with the number of sensors.
9. Figure 4 is hard to read. It is a bit confusing since the x-axis and y-axis only represent the condition where the measurements are done. Maybe presenting the results in a clearer way would be easier to follow. The S-shape actual trajectory in each sub-figure doesn't have any axes for example. It is not clear what do you mean by under-sampling when the speed is increased. It would be better to elaborate more on this.

Reviewer #3

(Remarks to the Author)
The paper by Weng et al presents photonic-integrated quantum sensor array based on optically detected magnetic resonance (ODMR) using nitrogen-vacancy (NV) centers in diamond for detection of microscale magnetic localization. The manuscript starts from the introduction of the photonic-integrated quantum sensor array, which has eight NV sensors. The concept of the photonic-integrated quantum sensor array shown in Fig. 1 is interesting, since in general setting the NV nanodiamonds at planed locations is difficult. The eight NV centers show nearly uniform ODMR spectra in Fig. 2, which guaranty small errors between eight NV sensors. Then, experimental data for a magnetized tip (a size of 30 μm) shows an average of 23 μm estimation error, which is within the tip size, but not so small. The experimental dynamical tracking of moving magnetic needle tip indicate a frame rate of several mHz is possible. Unfortunately, the photonic-integrated quantum sensor array was not applied for real microrobot system but performed under simulations. Even though, this manuscript presents an original instrument of photonic-integrated quantum sensor array. This manuscript might be suitable for the publication in Nature Communications if the authors add more discussion whether this photonic-integrated quantum sensor array can really be used for medical applications. More specific points (questions) are listed below.

1. The sensing area of the current photonic-integrated quantum sensor array is roughly 1 mm. The microrobot can travel along the blood tubes in their application (e.g., Ref. [17]), and therefore, it is necessary to connect several tens of NV arrays if we want to track the motion of the microrobot over 10 mm. In this case, it seems not so realistic to connect so many arrays.

2. In addition, the cw-ODMR requires a gold antenna to apply microwave field for NV centers. So, if the number of NV sensors is increased, the size of the antenna also increases. Please outline possible layout of the gold antenna if the number of NV sensors is increased.

3. The authors introduced the full 2D NV array could be used for sensing at the same time by replacing the 8-channel excitation fiber array with an eight-by-eight matrix fiber array (page 2). This is interesting, but how the each PL signal (8x8) can be separately detected through the eight waveguides?

4. Fig. 1d shows the image of nanodiamond array. Although the PL of the selected eight nanodiamonds (by the yellow rectangle) show nearly uniform ODMR signal (Fig. 2b), I am wondering if other nanodiamond array, e.g., the right next line of the yellow rectangle, also show nearly uniform ODMR signal. Please comment if this is so.

5. The spatial resolution of the photonic-integrated quantum sensor array is determined by the distance between the NV centers, which is ~125 µm (y-direction) in the present study and Ref. [15]. It seems that 100 nm diameter nanodiamonds can work in more tiny scales. Is it possible to make much smaller arrays to perform the sensing for nanorobot [Chem. Rev. Vol.121, 4999 (2021)] in the future?

6. In page 10, it is mentioned that microrobot's motion speed below 0.14 µm/sec is trackable using the photonic-integrated quantum sensor array. This means the microrobot can travel only ~0.5 mm/hour, which will be too slow to realize drug delivery. In the real applications, microrobot in blood tubes will move at a high speed of 600 µm/sec or higher than 1 mm/sec [e.g., Ref. (17) and Science Robotics vol.5, eaba5726 (2020)]. Is it possible to follow such a high-speed using photonic-integrated quantum sensor array with a frame rate of several mHz?

7. Under the application to microrobot (the position tracking of a microrobot in Fig. 5), the estimation error increases much larger than 50 µm when the microrobot moves away from the good operation area (-750 µm). If the speed of 600 µm/sec or higher than 1 mm/sec is applicable, it will be difficult to track the motion of microrobot. Please discuss this issue?

Overall, I feel this manuscript presents the new idea of photonic-integrated quantum sensor array based on cw-ODMR using NV centers in nanodiamonds. The current version is, however, not so appealing the broad range of readers since I am not sure if this photonic-integrated quantum sensor array can be used in real microrobot (and even nanorobot) applications. Probably, it will be possible to somehow improve the applicability of this array into biomedical system if the authors further discuss the layout of the photonic-integrated quantum sensor array.

Version 1:

Reviewer comments:

Reviewer #1

(Remarks to the Author)
In the updated manuscript and supplementary information, H.-C. Weng et al. have added a substantial number of changes and additions that have adequately clarified most of my questions and concerns. It is still a little disappointing that the most striking and impactful potential of the work (full translation and rotation tracking) is left to simulations and idealized arguments/estimates. I have a few more comments related to the author's additions.

Comments/concerns:
1. The tip motion arrows in figure 4d-f gives the impression that the tip is moving continuously and in an arbitrary way. I don't see why the tip motion should be displayed any differently than it is shown as in Fig. 1a-c.
2. Why not use higher order correlations to go beyond linear error scaling with the machine learning model? For N sensors and k subsets you would get ~N^k combinations (~N^2 for pairs, ~N^3 for triplets, etc.), which would lead to an error that scales as ~ N^(k/2). Is there some fundamentally unique property with pair information that disappears with larger group number in the machine learning model?
3. Many claims in the conclusion section rely on dramatic improvements in sensitivity. While ~1 nT/sqrt(Hz) has been demonstrated in some NV ensembles (refs. 28, 43), they use different experimental platforms. The manuscript and SI assume this can be achieved. How could a sensitivity improvement by 100000x realistically happen for the experimental setup in the manuscript? Even driving with 100x laser power (at saturation) and increasing coherence times by 10x with pulse protocols cannot take ~100 uT/sqrt(Hz) to ~1 nT/sqrt(Hz).

Reviewer #2

(Remarks to the Author)
I'd like to thank the authors for addressing my comments in the revised manuscript.

Reviewer #3

(Remarks to the Author)
I have read through both the revised manuscript and the response letter by Weng et al. I believe the authors address all the criticisms accordingly and they have improved the manuscript by considering the suggestions. In particular, the authors have added useful discussion for scalability of the photonic-integrated quantum sensor array. After the revision, the impact of this paper has been enhanced and its readability is now acceptable, so that the manuscript can now be recommended for the publication in Nature Communications.

Open Access This Peer Review File is licensed under a Creative Commons Attribution 4.0 International License, which permits use, sharing, adaptation, distribution and reproduction in any medium or format, as long as you give appropriate credit to the original author(s) and the source, provide a link to the Creative Commons license, and indicate if changes were made.
In cases where reviewers are anonymous, credit should be given to 'Anonymous Referee' and the source.
The images or other third party material in this Peer Review File are included in the article's Creative Commons license, unless indicated otherwise in a credit line to the material. If material is not included in the article's Creative Commons license and your intended use is not permitted by statutory regulation or exceeds the permitted use, you will need to obtain permission directly from the copyright holder.
To view a copy of this license, visit https://creativecommons.org/licenses/by/4.0/

Please find attached a point-to-point response (in black) to the useful comments and suggestions raised by the Reviewers (in blue). **Corresponding changes (in red) are indicated in the *quoted paragraph (in Italic).*** With the comments thoroughly addressed and the manuscript majorly revised, we feel that our manuscript has been much improved by all Reviewers' insightful comments and suggestions. We hope the work can be reconsidered for publication in Nature Communications.

Reviewer #1 (Remarks to the Author):

1. This work does not show a substantial technical advance and there is no fundamentally new science gained. This work is a follow-up to a previous work (Ref. 15, by the same authors) and the basic experimental principles and techniques are not significantly different; A MW antenna, improved optical coupling efficiency, and parallel sensor operation (similar to Refs. 7-14) are the main additions. The ~50 uT/sqrt(Hz) sensitivity and ~1 mT dynamic range are not remarkable compared to existing literature. Additionally, the use of machine learning for magnetic tracking with NV centers is not a new idea (see Meneses et al. 2025 doi.org/10.48550/arXiv.2502.14683).

We respectfully disagree that this work is a follow-up of Ref 15. We believe this view overlooks the distinction between the fabrication method and the scientific function. Our previous work (Ref. 15, *ACS Photonics*) was a materials science study focused on the heterogeneous integration of diamond on silicon. It produced a coupled diamond-SiN device to prove manufacturability. The current manuscript is a quantum systems engineering study. It addresses a fundamentally different set of challenges: cross-talk, uniform array addressing, and algorithmic reconstruction from distributed sensors. To address the novelty concern directly, we add text contrasting the present work with our ACS Photonics work (Ref. 15) in terms of scientific question and capability.

Introduction, third paragraph: ***Compared to the bulk-optical methods… Existing integrated diamond platforms have largely focused on the manufacturing challenges of coupling emitters to waveguides, such as via heterogeneous integration [15]. However, the realisation of a practical quantum sensor requires more than just integration; it demands a scalable architecture for information extraction, routing excitation and collection light to multiple sites simultaneously with minimal crosstalk and uniform efficiency.***

A single sensor (Ref. 15) cannot triangulate a moving source in real-time; it requires the target to be static while the sensor is mechanically scanned. Our array architecture solves this by acquiring data from multiple geometric vantage points simultaneously, the parallel sensor operation. This is a qualitative change in capability, not a quantitative increment. We have revised the Introduction to more precisely state what is new here.

Introduction, fourth paragraph: ***In this work, we present an operational eight-channel sensor array that bridges the gap between device physics and system-level applications. By performing Continuous-Wave Optically-Detected-Magnetic-Resonance (CW ODMR) measurements, the eight sensors record spatially-resolved magnetic fields simultaneously, allowing multi-point field reconstruction with continuous-time signal processing and direct application in magnetic localisation.***

Introduction, fourth paragraph: ***We show that this readout, when combined with a neural-network reconstruction algorithm, solves the inverse localisation problem for dynamic magnetic targets, a capability inaccessible to single-sensor devices. Specifically,*** **we estimate the static position…**

Introduction, fourth paragraph: ***As an application-motivated case study, we simulate microrobot-relevant magnetic profiles [16] to quantify the requirements under which translation and rotation tracking become feasible, noting that rotation tracking requires vector information available in the single-NV (or oriented-ensemble) regime. In simulation with…***

Introduction, fourth paragraph: ***More broadly, this architecture addresses the interconnect bottleneck for scaling multi-NV protocols beyond the optical table by shifting multiplexing from bulk optics to guided-wave routing and detector-channel readout, enabling deployable localisation in optically inaccessible environments such as in in vivo [17] or microfluidic [18] scenarios.***

We note that Refs 7–14 achieve parallel operation primarily through bulk-optic wide-field microscopy (i.e., using cameras/SLMs). While EMCCD cameras offer parallelism, they rely on macroscopic free-space optics and are limited in frame rate. In contrast, chip-scale integration with fast detectors offer low latency and fast feedback. Our work demonstrates parallelism within the integrated waveguide architecture**.** Achieving this required solving the 'interconnect bottleneck'—routing multiple optical channels on a chip with high isolation and uniform collection efficiency. This is a photonic engineering challenge distinct from imaging a diamond with a camera and is essential for the transition from optical-table experiments to portable, integrated devices. We revise the abstract as follows:

Abstract: ***We use simulations of microrobot-relevant magnetic profiles as an application-motivated case study to quantify the operating bounds and requirements for translation and rotation tracking. By moving multi-NV localisation from bulk-optical addressing to a fibre-addressed, guided-wave, multi-channel architecture with simultaneous distinct readout, this work provides a scalable architecture towards scalable magnetic localisation in optically inaccessible environments.***

We thank the Reviewer for bringing Meneses' work to our attention (Meneses et al. 2025 doi.org/10.48550/arXiv.2502.14683). We now reference this work (Ref. 48) noting where their application to metre-scale tracking is different:

Conclusion, last paragraph: ***The deterministically positioned NVs also allow static control with fibre arrays and waveguides, in contrast to stochastic sensors that need to be addressed by fast programmable optics [11,12]. Machine-learning-based localisation, similar to what was found in [48], extends to micrometre-sized objects.***

2. I do not believe the experiments demonstrated support the claims that this platform can be used for dynamic position and rotation tracking of magnetic objects with an array of single NV sensors. The magnitude-based NV ensembles cannot track rotations, and I do not see how nanodiamond drop casting will repeatably result in arrays of single NV centers. Moreover, the random orientation of each NV center would need to be calibrated before use. The author should demonstrate parallelized sensing with single-NV arrays to suggest the feasibility of position and rotation tracking.

We address the Reviewer's three concerns in turn: single-NV feasibility on this platform, repeatability of array fabrication, and NV-axis calibration. First, we wish to highlight that we have experimentally demonstrated both CW ODMR and coherent spin control (Rabi oscillations) of a single NV centre on this photonic platform (Supplementary Section G). This confirms that the architecture is compatible with single-NV operation. The barrier to operating all eight sites with single NVs simultaneously is the available pump laser power: single-NV sites must be driven at saturation, requiring 10-100x the power used for the ensemble measurements presented here. This is currently an equipment constraint that we make explicit in our revision.

Secondly, regarding the fabrication of single-NV arrays, we add a quantitative feasibility analysis based on repeatability data for the positioning process. Specifically, Supplementary Section A shows a 97.5% positioning yield (78/80 sites). Using the single-NV occupancy statistics reported for the same nanodiamond type and platform in Ref. 15 (modelled as a Poisson occupancy with mean $\lambda \approx 0.58$, giving $P(0) \approx 0.56$, $P(1) \approx 0.33$, and $P(\geq 2) \approx 0.11$), the effective probability of obtaining a single NV at a positioned site is ≈0.32; the remaining outcomes are empty/no-NV sites and multi-NV sites. We have added Supplementary Section I to propagate these per-site probabilities to an eight-site array and shows how repeat deposition/screening on empty sites increases the likelihood of obtaining an eight-site single-NV array (e.g., allowing up to four deposition attempts corresponds to an expected ~1 successful eight-site array when screening on the order of 25–30 arrays).

We agree with the Reviewer that parallel single-NV tracking is the ultimate goal for this technology. The novelty of this work lies in the scalable photonic architecture, which functions regardless of the number of NVs per site. We have tightened the main text to clearly separate ensemble-sensor experimental results from the single-NV roadmap. We also note that for a *non-uniform* microrobot with a defined magnetic asymmetry, partial orientation information (such as out-of-plane tilt when the heading direction is set by the driving field) may in principle be accessible from magnitude measurements alone, and is an interesting direction for future work.

Third, we agree with the Reviewer’s point that single NV centres should be calibrated before use to identify the NV axis. This was considered in the Fig.5d and 5e simulations, with single NV centres in different orientations. We have added additional comments in Supplementary Section H. Along with the simulations in Fig. 5, we hope the feasibility is now clarified.

Supplementary Section H, last paragraph: ***For position and orientation tracking with single NVs… To experimentally realise magnetic localisation with single-NVs, it is necessary to characterise the single-NV axes, for example, following [6]. In our current setup, the amount of pump laser power required to enter the saturation regime (estimated 10 to 100 times) prevents us from implementing parallel single NV sites.***

3. Machine learning methods require massive amounts of training data that i) is highly sensitive to the magnetic field of the object and ii) includes knowledge of the object’s position during measurement (which is already the point of the measurement). How do the authors suggest conducting tracking experiments, if the position, orientation, and type of magnetic object is unknown from the start?

We thank the Reviewer for raising the important point. Our objective is to demonstrate tracking with a chosen type of magnetic object. In order for it to be used in microrobotic applications, training of the model can be first performed in a testbed (such as with a medical phantom, see new Ref. 35) where microrobot positions can be monitored. Because the features of the microrobot are learned by the model, the same type of microrobots can then deployed for mission in optically inaccessible environments. We have clarified this point in:

Fig. 5, Captions: ***a. Targeted application scenario…Note that the machine learning model can be first trained outside the test body [35]. The same type of microrobots can then deployed in optically inaccessible environments. Also, magnetic field used …***

4. With parallelized multi-NV measurements, theoretical and practical scalability should be addressed. The authors claim this platform is scalable, but no discussion about scalability is given. What potential bottlenecks do the authors foresee (low yield of single-NV nanodiamonds, size resolution for the excitation fiber array, etc.)?

We thank the Reviewer for the comment. We have now included discussion on system scalability and challenges for single NV sensors and the photonic scheme. We include these in the Supplemental Section I. Corresponding changes are also made in the main text and a new paragraph is added in the conclusion section.

Beyond the single-NV calculation in point 2, we have also now included scalability discussion in the Conclusion.

Section- Magnetic microrobot tracking-simulations and the use of single-NV Sensors, last paragraph: ***Single NVs have been integrated in similar platforms [15] and we also show that CW ODMR and coherent spin operation (Rabi oscillations) is possible experimentally (see Supplementary Section G). An array of single-NV sensors can be efficiently created (as shown in Supplementary Section I).***

Conclusion, fourth paragraph: ***Looking forward … Notably, the system-level advance demonstrated here---simultaneous, distinct, multi-channel readout of spatially separated NV sensors on a fibre-addressed photonic platform---provides a scalable hardware foundation (requiring photonic mode engineering similar to [42]) on which such sensing-protocol improvements can be deployed.***

Conclusion, fifth paragraph: ***The sensor array is also scalable. For example, one can extend the operation range by (a) increasing the number of sensors in the current line and (b) adding a parallel array to the current one. The enlarged array can be controlled with commercial fibre arrays, with up to 96 fibres and the same 127 µm spacing. This could extend the operation Y range in Fig. 5c by more than ten times to ~ 15 mm. A second parallel array, on the other hand, doubles the operation X range in Fig.5c. On top of that, by improving the sensor sensitivity to around 1 nT/$\sqrt{Hz}$ level, one can simultaneously increase the frame rate by 10^4 times and the operation range by another 10 times (see Supplementary Section I for detailed calculations). Spin addressing over an extended area (at 10 mm scale) can be achieved with specially designed microwave antennas [22,43,44]. Notably, with the number of sensors significantly increased, machine learning based methods become the only***

way to efficiently extract the correlations between sensor readouts for magnetic inverse calculation. In contrast, to track smaller magnetic robots (such as nanowire robots of overall length 5-10 μm [45]), sensor arrays with spacing ten times smaller (around 10-20 μm) can be achieved by bringing the parallel waveguides closer in the sensor region and optical excitation with multi-core fibres [46].

5. The authors should include a comparison to the works of S. Chen et al. 2021 (doi.org/10.1073/pnas.2118876119) and N. Mathes et al. 2024 (doi.org/10.1039/D3NA00684K) that images micro/nano-sized magnetic particles inside a biological sample with NV centers.

We thank the Reviewer for highlighting these relevant studies. We have now added citations to Chen et al. and Mathes et al. and clarified how they relate to our work. Chen et al. and Mathes et al. demonstrate widefield NV-based magnetic imaging of magnetic particles in biological specimens with direct optical access. In contrast, our work demonstrates a fibre-addressed, photonic-integrated multi-sensor array with guided-wave routing and distinct detector-channel readout, and we use the resulting multi-point measurements to solve an inverse localisation problem without requiring direct optical access to the target. We now discuss this distinction and complementarity in the revised manuscript.

Section-Static magnetic localisation through multi-point field reconstruction, Second paragraph: ***Through multi-point field reconstruction... Magnetic localisation, different from direct wide-field imaging [31,32], (1) does not require direct optical access to the target (since the target's field is recorded remotely by the sensors), and (2) has ultimate resolutions fundamentally limited only by the physical size of the sensors (similar to the localisation method used in [7])...***

6. The authors refer to eight NV sensors in the text. However, each sensor is not an NV center but an ensemble of many nanodiamonds containing multiple NV centers. The authors should clear up this ambiguity throughout the abstract and text.

We thank the Reviewer for the reminder. We have now mentioned the ensemble sensors explicitly in the abstract and the text.

Abstract: ***In this work, by integrating NVs with foundry silicon-nitride photonic integrated circuits, we realise the scalable operation of eight localised NV-ensemble sensors in an array, with simultaneous, distinct readout of the individual sensors.***

Fig. 2 Captions: ***The PL signal of the eight NV-ensemble sensors collected from each waveguide when the excitation (exc.) fibre array is scanned…***

Fig. 3 Captions: ***a. Image showing the magnetic needle tip above the chip… The NV-ensemble sensors characterised in Fig. 2 are used.***

7. Just how far below optical saturation do the NV ensembles operate? Are saturation curves available?

We operate each sensor at <0.2 mW pump power per site (measured at the excitation-fibre output), i.e. <5% (~20X lower) than the saturation power (> 4 mW) reported in Ref. 15,

where saturation curves are provided. The operating point is ultimately limited by SPAD-based readout; higher pump powers would be compatible with photodiode detection (and for the eight-channel readout, require higher power lasers than available for this experiment). We have now included the discussion as follows.

Section- Parallel operation of eight NV magnetic sensors, last paragraph: ***The full operating window of the sensors… we operate with <0.2 mW pump power per site, below the saturation power (>4 mW as shown in [15]), one can further increase the sensitivity (decrease $\eta_{CW-ODMR}$) by pumping the sensors harder and potentially detecting the signal with low-noise photodiodes as opposed to single-photon detectors.***

8. Why does adding more sensors improve the reconstruction linearly in figure 3f? A naïve assumption would be that it improves proportional to the square root of the sensors.

We thank the Reviewer for the opportunity to clarify. The scaling between the number of sensors and estimation error (originally Fig. 3f, now Fig. 3h) can be understood from the $1/\sqrt{N}$ scaling for data set (original Fig. 3h, now Fig. 3g). The useful data for the machine learning model is the number of pair-wise correlations (that scales with n^2) that can be extracted from n sensors, similar to the mathematics principle behind the correlated sensing or covariance sensing works (cite). As a result, the $1/\sqrt{n^2}$ becomes an inversely linear correlation. To improve the flow, we have shifted the orders of the subfigures in Fig. 3.

Fig. 3 captions: ***Fig. 3 Experimental magnetic localisation with multi-point field reconstruction. f The $1/\sqrt{t}$ scaling of the average estimation error with the integration time t. g Relationship between average estimation error and training dataset size N, showing $1/\sqrt{N}$ scaling. Measurements are done with a 360 s integration time. h The linear decrease of averaged estimation error with increased number of sensors used simultaneously. The full training data set and a 360-s integration time are used.***

The following clarifications are also included.

Section- Static magnetic localisation through multi-point field reconstruction, second last paragraph: ***The estimation error is affected by various experimental parameters. For example, by integrating the ODMR measurements for a shorter period of time, the localisation estimations can be made faster, however, at a higher noise level. We show the scaling between integration time t and estimation error in Fig. 3f, which matches a $1/\sqrt{t}$ fitting as expected for the shot noise in photon counting. To see how much training data is needed for machine-learning based magnetic localisation, we analyse how the averaged estimation error scales with the dataset size N in Fig. 3g. The estimation error follows $1/\sqrt{N}$ scaling. This is expected since, with fixed operation area A, $\sqrt{A/N}$ presents the resolution of sampled data points (with length unit). Using more sensors for magnetic localisation allows us to collect more data simultaneously, which improves the reconstruction of the magnetic field. This advantage is captured by Fig. 3h, showing the estimation error reduces linearly with the number of sensors used. To understand the linear relation, one should first note that the useful information for the machine learning model is the number of pair-wise correlations (which scales with n^2) that can be extracted from n sensors, similar to the***

mathematical principle behind correlated and covariance sensing [7,9]. As a result, the 1/$\sqrt{N}$ scaling for data sizes becomes 1/$\sqrt{n^2}$=1/n for the number of sensors.

9. In figure 4, the tip is not moving at the indicated speed, but instead takes discrete steps every X seconds. The authors should mention that measurements without smearing present an idealized version of motion tracking and should change “tip moving speed” to “effective tip speed”.

We thank the Reviewer for the great suggestions. We have now explicitly mention that the tip motion is in discrete steps and the presented speed is effective speed. The effect of smearing is illustrated in more details.

We have also improved the presentation of Fig. 4 and corresponding paragraphs with suggestions from Reviewer 2, point 9. The figure is updated and the original figure is moved to Section F in the supplemental material. The following changes to the main text are made.

Fig. 4 Captions: ***The needle tip, when programmed to move at different speeds (effective speeds as calculated by step size divided by the dwell time), is tracked with different frame rates for comparison. a-c compare the tracking at different tip moving speeds, while d-f present tracking with different frame rates. Note that the same machine-learning model is used for magnetic localisation and the trajectory falls within the operation area in Fig. 3a. Note that a and d show the same data. The deviation of the reconstructed (rec.) trajectory from the true trajectory is quantified by the Mean Perpendicular Distance (MPD), which increases for higher moving speeds due to under sampling (tip moved multiple steps in one frame) and higher frame rates due to shot noise, as illustrated by the diagrams.***

Section- Dynamics of a moving magnetic source: continuous-time tracking: ***The results are shown in Fig. 4, with the red curve showing the true trajectory and the connected data points giving the reconstructed trajectory. When the needle tip speed increases and the frame rate is fixed, we observe a smeared trajectory due to under sampling (tip moved multiple steps within one frame/measurement). For example, a larger deviation from the true trajectory can be seen in Fig. 4c, especially where the curvature is large, compared to Fig. 4b and Fig. 4a. On the other hand, comparing Fig. 4d, Fig. 4e, and Fig. 4f, when the needle tip moves at a certain speed, a noisier trajectory is observed at higher frame rates (as expected from Fig. 3g due to shot noise). To quantify the fidelity of the trajectory reconstruction, we calculate the error in terms of the Mean Perpendicular Distance (MPD), i.e., the averaged shortest distance from each estimated data point to the true trajectory. Note that the stationary averaged estimation error is not used as the metric here because the movement is typically not synchronised with the ODMR measurement integration time in tracking unknown dynamics (i.e. the magnetic localisation can be performed while the tracked object moves, depending on the frame rate chosen). We can observe an increased MPD for under sampling in Fig. 4c (due to smearing when the integration time is longer than the dwell time). In Fig. 4e and Fig. 4f, the MPD also increases due to shot noise. Supplementary Section F visualises both effects at the same time with MPD calculated.***

10. In figure 5b, the authors should consider a contour line to show the < 50 um region, or use multiple contours, rather than a box to denote their "good operating area".

We appreciate the suggestions. We have now replaced the box with a contour line and modified the caption for Fig. 5b.

Fig. 5 Captions: ***b Error map of simulated microrobot magnetic localisation, with eight ensemble NV sensors. The robot-position-dependent estimation error is plotted with the relative positions of the NV ensemble sensors labelled. The good operation area (with estimation error*** $\leq 50\mu m$***) is shown by the contour line.***

Section- Magnetic microrobot tracking- simulations and the use of single-NV Sensors, second paragraph: ***For microrobot tracking applications…Within a good operation area of 750*** μm ***by 1400*** μm ***(as shown by the contour line), we observe*** $\leq 50\mu m$ ***estimation error.***

11. For completeness, the authors should include a supplemental figure with the ~ 750 tip positions used in figure 3d-f for reference. Additionally, they should show the separation of that data into the training/validation subsets that produced figure 3h.

We have now presented the dataset used in Fig. 3 in the new supplementary section (Section E). We show the train-validation-test separation in the new Fig. S4. Corresponding changes in the main text can be found as follows.

Method Section- Machine learning methods for experimental magnetic localisation, second paragraph: ***The labelled data is split such that 90% is used for training, 7% is used for validation while training, and 3% is used for testing and visualisation in Fig. 3d. The train-validation-test separation of the dataset is shown in Supplementary Section E.***

12. Typos/mistakes:

1. "TE-mode" should be written out fully when first introduced.
2. Refs. 10 and 13 should be updated, as both are now published.
3. The color bar labels are missing in supplementary figures 2, 3, and 4.

We thank the Reviewer for identifying the typo and mistakes. We have made the following improvements.

Section- Scalable NV quantum sensing with photonic integrated circuits, first paragraph: ***The PL signal is evanescently coupled to the single transverse electric (TE)-mode waveguides [15] and sent off-chip to a second 8-channel collection fibre array through edge couplers…***

The original Ref. 10 and Ref. 13 have been updated. Colour bars in supplementary figures are now added. We also spotted other typos/writing mistakes and made the following improvements.

Introduction, second paragraph: ***However, widefield ODMR with EMCCD cameras is constrained by frame rates with integration time*** $\geq$ ***10 ms, which is much slower than the NV spin readout time (300 ns).***

Method section- Machine learning methods for experimental magnetic localisation, second paragraph: ***Once trained, the same model can be used for magnetic localisation as long as experimental conditions are maintained (the magnetic needle tip setup and orientation).***

Fig. 1c Captions: ***c The silicon-nitride photonic chip… The middle red-shaded area is unclad for nanodiamond positioning.***

Section- Parallel operation of eight NV magnetic sensors, last paragraph: ***The full operating window of the sensors… while the ODMR contrast decreases significantly to ≤1% for |B|> 2.2 mT.***

Section- Magnetic microrobot tracking- simulations and the use of single-NV sensors, last paragraph: ***The single NV sensitivity can degrade under unaligned fields due to spin mixing and quenching [38] but less significantly in the low field regime (< 5 mT) compared to NV ensembles (as shown in Fig. 2f).***

Method Section- Experimental setup: ***Here we include details of the experimental setup. … The NV centres are pumped by a 40-mW CW Cobolt 06-MLD laser. The NV spins are driven by a microwave source (SMB100AP20 Rohde & Schwarz)…***

Supplementary Section G, first paragraph: ***With $<$4 mW pump power used, we observe the photoluminescence counts around 6000/s recorded from one end of the waveguide…***

Reviewer #2 (Remarks to the Author):

1. How much is the green pump rejection in the experiment? It would be helpful to quantify this.

We would like to thank the Reviewer for the useful comment. We believe the pump rejection is best quantified by our previous work (Ref. 15) which uses the same waveguide and nanodiamond positioning material platform. It is shown by the orthogonal pump-probe scheme provides a −70 dB attenuation to the pump coupling to the waveguide. We have now included the discussion in the manuscript.

Section- Scalable NV quantum sensing with photonic integrated circuits, first paragraph: ***Note that by coupling the NV dipole emission to single mode waveguides, this helps reject fluorescence from the excitation fibre (up to -70 dB as shown in [15]) …***

2. Is there a reason to place the nanodiamond area in the middle of the Silicon-nitride waveguides compared to one of the ends?

We thank the Reviewer for raising the point. For our experiments, the nanodiamond area is placed in the middle of the chip for ease of operation and symmetricity of the setup. In principle, one can also place it at the ends with appropriate changes to the setup. We have now added a clarification sentence in the manuscript.

__Fig. 1c Caption__*: The silicon-nitride photonic chip… The middle red-shaded area is unclad for nanodiamond positioning. The nanodiamond area is placed in the middle of the chip for ease of operation and symmetricity. The edge couplers (enclosed by the yellow dashed-line box) are fabricated with a deep etch to ensure surface smoothness.*

3. For the diamond deposition, do you remove the PMMA mask after deposition? In the text, it is mentioned that "residual nanodiamonds on the PMMA mask are then removed by acetone", does this mean acetone could remove the wanted deposited nanodiamonds? Also, what is the repeatability of this technique?

We thank the Reviewer for pointing out the ambiguity. The unwanted nanodiamonds on the PMMA mask is removed during the lift-off of PMMA in acetone. Only the nanodiamonds attached to the waveguides over the patterned sites remain. We have now clarified this in the manuscript.

__Method section- Nanodiamond positioning:__ *The dropcasting is repeated five times to ensure proper coverage over the area. The residual nanodiamonds on the mask are removed during the lift-off of PMMA in acetone, followed by a gentle ultrasonication for 10 seconds. Only the nanodiamonds attached to the waveguides over the patterned sites remain.*

To show the repeatability of this technique, we now have included an image in the supplementary (Fig. S1) in the new section (Section A), showing near-unity yield on a different chip with an even larger array of nanodiamonds. Corresponding changes are made in the manuscript.

__Section- Scalable NV quantum sensing with photonic integrated circuits, second paragraph:__ *We realise… The FNDs, each hosting more than 1000 NVs, are selected to guarantee near-unity NV yield per nanodiamond site and provide a strong optical signal. We show the repeatability of the method in Supplementary Section A.*

4. It would be beneficial for the reader to explain how Fig 1g is calculated. Is it measured or simulated data? Does it consider the coupling efficiency of the NV fluorescence to the waveguides as well or just the waveguides to the collection fibers? Please elaborate in the manuscript. Waveguide-to-chip coupling is defined as 85%, however in Fig. 1g the coupling efficiency is around 25% maximum. In addition, is waveguide-to-chip coupling the same as fiber to on-chip waveguide coupling?

We thank the Reviewer for this opportunity to clarify. The blue solid line in Fig. 1g presents the normalised NV spectrum, simulated following Ref.19. The normalised spectrum is multiplied by the experimentally measured waveguide-to-fibre efficiencies for edge couplers and grating couplers and shown by the green- and yellow- shaded areas, respectively. To clarify this, we have made the following change.

__Fig. 1g Captions:__ *Comparison of the coupling efficiencies over spectrum for edge couplers and grating couplers. The normalised NV spectrum (blue, simulated following [19]) is compared to those multiplied by the waveguide-to-fibre efficiencies*

for edge couplers (green) and grating couplers (yellow). Wavelength abbreviated Wl. in the plot.

The legend in Fig. 1g is also improved for clarity. We also thank the Reviewer for pointing out the unclear sentence. We mean that the waveguide-to-fibre coupling remains up to 85% of its original efficiency with eight channels coupled in parallel. We have clarified this in the updated manuscript.

Section- Parallel operation of eight NV magnetic sensors, first paragraph: ***Parallel operation of the eight NV ... Note that the waveguide-to-fibre coupling remains at up to 85% of its original efficiency (as shown in Fig. 1g) with eight channels coupled in parallel.***

To avoid confusion, we have now used the terms *waveguide-to-fibre couplings* throughout the manuscript.

5. The authors mentioned "the physical size of the sensor (and thus the ultimate spatial resolution for quantum sensing) is defined directly by the deposition area (which is 4 μm by 500 nm)." Is it not defined by the fiber array pitch (127 um)? The sensor can't resolve such small resolution that is claimed in the manuscript.

We believe this point arises from our imprecise statement that conflated the physical sensor volume (set by the nanodiamond deposition footprint) with the localisation precision of the inverse problem. The fibre-array pitch (127 μm) sets the sensor spacing / array geometry, but it does not by itself determine the achievable localisation error.

We revised the manuscript to (i) state that the deposition area defines the interaction volume (spatial averaging scale of the local field measurement), and (ii) clarify that localisation precision is governed primarily by SNR, local field gradient, and array geometry, consistent with Eq. 2. Corresponding changes in the manuscript are:

Section- Parallel operation of eight NV magnetic sensors, first paragraph: ***While the spot sizes… the physical size of the sensor volume (interaction region) is defined by the deposition area (4 μm by 500nm).***

Section- Static magnetic localisation through multi-point field reconstruction, last paragraph: ***The position estimation error, symbolising the practical magnetic localisation resolution, is determined by factors beyond the physical size of the senso r (a few μm in our case), such as the sensor sensitivity and the local magnetic field gr adient.***

Unlike imaging the object directly through camera where the density of pixels in a given area (or effectively the pixel-to-pixel distance) decides the spatial resolution, the magnetic localisation technique, similar to triangulation method used in Ref.7, has its ultimate resolution determined by the physical size of the sensor. That is, a change in the sensors' read is indistinguishable in any case when the object is moved by a distance smaller than the size of the sensor. The practical resolution is affected by other factors (such as the sensitivity and magnetic profile of the target) as summarised in Eq. 2. To further clarify, we have also made the following changes to the manuscript.

Section-Static magnetic localisation through multi-point field reconstruction, Second paragraph: ***Through multi-point field reconstruction... Magnetic localisation, different***

from direct wide-field imaging [31,32], (1) does not require direct optical access to the target (since the target's field is recorded remotely by the sensors), and (2) has ultimate resolutions fundamentally limited only by the physical size of the sensors (similar to the localisation method used in [7])...

6. The statement "the average slope is 22 ± 3 MHz/mT, which is about half the maximum splitting (2 × 28 MHz/mT) due to the averaged response of isotropic NV orientations" is not very clear. Is there a prior experiment in the literature with such performance? Does it make sense that the average response of the nanodiamonds changes the effective gyromagnetic ratio this much?

We thank the Reviewer for the comment. We note that the reduced effective gyromagnetic ratio is similarly observed in Ref. 23 and Ref. 24. In Ref. 24, it is characterised differently by an empirical equation with linear splitting 1.58 MHz/G (15.8 MHz/mT) and a quadradic term 0.17 MHz/G^2. The reduced effective gyromagnetic value is also supported by our simulations shown in Supplementary Section C. We have now clarified this in the manuscript.

Section- Parallel operation of eight NV magnetic sensors, third paragraph: ***The average slope* is 22 ± 3 MHz/mT *due to the averaged response of isotropic NV orientations* *(see derivation in Methods and similar simulation results in Supplementary Section C). The reduced effective gyromagnetic ratio for NV ensembles in FND is similarly observed in [23, 24].***

Supplementary Section C:
The Zeeman splitting is in the linear regime for |B|≥0.2 mT (with a slope of 26.9 MHz/mT).

7. It is not clear why the sensitivity doubled when magnetic field changes from 1mT to 1.5mT. This sounds like a very big change.

We appreciate this comment. $\eta_{CW-ODMR}$ doubles when the field increase from 1 mT to 1.5 mT due to ensemble broadening and reduced resonance contrast as shown in Fig. 2c and Fig. 2d. This is also captured by the numerical model presented in the method section (NV ensemble simulations). Due to the increased $\eta_{CW-ODMR}$, we have also concluded that the sensors operates best from 0.2 mT to 2.2 mT (see Supplementary Section C). We have now clarified this in the corresponding sentence.

Section- Parallel operation of eight NV magnetic sensors, third paragraph: ***Under 1 mT magnetic field strength, $\eta_{CW-ODMR}$ increases to $\geq$ $50\ \mu T/\sqrt{Hz}$ and doubles again to $\geq$ $100\ \mu T/\sqrt{Hz}$ under 1.5 mT due to increased ensemble broadening and reduced resonance contrast, as shown in Fig. 2c and Fig. 2d.***

8. It would be helpful for the reader to elaborate more on why the estimation error of the magnetic localisation experiment reduces linearly with the number of sensors.

We thank the Reviewer for the opportunity to clarify. The scaling between the number of sensors and estimation error (originally Fig. 3f , now Fig. 3h) can be understood from the $1/\sqrt{N}$ scaling for data set (original Fig. 3h, now Fig. 3g). The useful data for the machine

learning model is the number of pair-wise correlations (that scales with n^2) that can be extracted from n sensors, similar to the mathematics principle behind the correlated sensing or covariance sensing works (cite). As a result, the 1/$\sqrt{n^2}$ becomes an inversely linear correlation. To improve the flow, we have shifted the orders of the subfigures in Fig. 3.

Fig. 3 caption: ***Experimental magnetic localisation with multi-point field reconstruction. f The 1/$\sqrt{t}$ scaling of the average estimation error with the integration time t. g Relationship between average estimation error and training dataset size N, showing 1/$\sqrt{N}$ scaling. Measurements are done with a 360 s integration time. h The linear decrease of averaged estimation error with increased number of sensors used simultaneously. The full training data set and a 360-s integration time are used.***

The following clarifications are also included.

Section- Static magnetic localisation through multi-point field reconstruction, second last paragraph: ***The estimation error is affected by various experimental parameters. For example, by integrating the ODMR measurements for a shorter period of time, the localisation estimations can be made faster, however, at a higher noise level. We show the scaling between integration time t and estimation error in Fig. 3f, which matches a 1/$\sqrt{t}$ fitting as expected for the shot noise in photon counting. To see how much training data is needed for machine-learning based magnetic localisation, we analyse how the averaged estimation error scales with the dataset size N in Fig. 3g. The estimation error follows 1/$\sqrt{N}$ scaling. This is expected since, with fixed operation area A, $\sqrt{A/N}$ presents the resolution of sampled data points (with length unit). Using more sensors for magnetic localisation allows us to collect more data simultaneously, which improves the reconstruction of the magnetic field. This advantage is captured by Fig. 3h, showing the estimation error reduces linearly with the number of sensors used. To understand the linear relation, one should first note that the useful information for the machine learning model is the number of pair-wise correlations (which scales with n^2) that can be extracted from n sensors, similar to the mathematical principle behind correlated and covariance sensing [7,9]. As a result, the 1/$\sqrt{N}$ scaling for data sizes becomes 1/$\sqrt{n^2}$=1/n for the number of sensors.***

9. Figure 4 is hard to read. It is a bit confusing since the x-axis and y-axis only represent the condition where the measurements are done. Maybe presenting the results in a clearer way would be easier to follow. The S-shape actual trajectory in each sub-figure doesn't have any axes for example. It is not clear what do you mean by under-sampling when the speed is increased. It would be better to elaborate more on this.

We thank the Reviewer for the suggestion. We have improved the presentation for Fig. 4, with the subfigure x and y axes labelled. The concept of under sampling and effect of shot noise are also illustrated with diagrams. Part of the data presented in the original Fig. 4 is now moved to supplementary (Section F) for clearness and completeness. Corresponding changes are also made in the manuscript for the updated Fig. 4 presentation.

Fig. 4 Caption: ***The needle tip, when programmed to move at different speeds (effective speeds are calculated from step size divided by the dwell time), is tracked***

with different frame rates for comparison.** a-c compare the tracking at different tip moving speeds, while d-f present tracking with different frame rates. Note that the same machine-learning model is used for magnetic localisation and the trajectory falls within the operation area in Fig. 3a. Note that a and d show the same data.* ***The deviation of the reconstructed (rec.) trajectory from the true trajectory is quantified by the Mean Perpendicular Distance (MPD), *which increases for higher moving speeds due to under sampling (tip moved multiple steps in one frame) and higher frame rates due to shot noise, as illustrated by the diagrams.*

Section- Dynamics of a moving magnetic source: continuous-time tracking: ***The results are shown in Fig. 4, with the red curve showing the true trajectory and the connected data points giving the reconstructed trajectory.*** *When the needle tip speed increases and the frame rate is fixed, we observe a smeared trajectory due to under sampling (tip moved multiple steps within one frame/measurement). For example, a larger deviation from the true trajectory can be seen in Fig. 4c, especially where the curvature is large, compared to Fig. 4b and Fig. 4a. On the other hand, comparing Fig. 4d, Fig. 4e, and Fig. 4f, when the needle tip moves at a certain speed, a noisier trajectory is observed at higher frame rates (as expected from Fig. 3g due to shot noise).* ***To quantify the fidelity of the trajectory reconstruction, we calculate the error in terms of the Mean Perpendicular Distance (MPD), i.e., the averaged shortest distance from each estimated data point to the true trajectory. Note that the stationary averaged estimation error is not used as the metric here because the movement is typically not synchronised with the ODMR measurement integration time in tracking unknown dynamics (i.e. the magnetic localisation can be performed while the tracked object moves, depending on the frame rate chosen).*** *We can observe an increased MPD for under sampling in Fig. 4c (due to smearing when the integration time is longer than the dwell time). In Fig. 4e and Fig. 4f, the MPD also increases due to shot noise. Supplementary Section F visualises both effects at the same time with MPD calculated.*

Reviewer #3 (Remarks to the Author):

1. The paper by Weng et al presents photonic-integrated quantum sensor array based on optically detected magnetic resonance (ODMR) using nitrogen-vacancy (NV) centers in diamond for detection of microscale magnetic localization. The manuscript starts from the introduction of the photonic-integrated quantum sensor array, which has eight NV sensors. The concept of the photonic-integrated quantum sensor array shown in Fig. 1 is interesting, since in general setting the NV nanodiamonds at planed locations is difficult. The eight NV centers show nearly uniform ODMR spectra in Fig. 2, which guaranty small errors between eight NV sensors. Then, experimental data for a magnetized tip (a size of 30 μm) shows an average of 23 μm estimation error, which is within the tip size, but not so small. The experimental dynamical tracking of moving magnetic needle tip indicate a frame rate of several mHz is possible. Unfortunately, the photonic-integrated quantum sensor array was not applied for real microrobot system but performed under simulations. Even though, this manuscript presents an original instrument of photonic-integrated quantum sensor array. This manuscript might be suitable for the publication in Nature Communications if the authors add more discussion whether this photonic-

integrated quantum sensor array can really be used for medical applications. More specific points (questions) are listed below.

We thank the Reviewer for their positive assessment of our instrumental originality.

2. The sensing area of the current photonic-integrated quantum sensor array is roughly 1 mm. The microrobot can travel along the blood tubes in their application (e.g., Ref. [17]), and therefore, it is necessary to connect several tens of NV arrays if we want to track the motion of the microrobot over 10 mm. In this case, it seems not so realistic to connect so many arrays.

We thank the Reviewer for the comment. Extending the sensing range (the Y range in Fig. 5b) to over 10 mm is believed achievable, considering 80 sensors in an array with the same 0.127 mm spacing used in the current experiments (79 x 0.127 mm =10.033 mm). This could readily be achieved with a larger chip. Using the same photonic scheme (Fig. 1), the sensor control can be realised with fibre arrays commercially available (Oz optics, up to 96 fibres in an array with 127 mm pitches) for NV optical excitation and PL collection from edge couplers. To illustrate the scalability, we have now included a new paragraph in the conclusion section.

Conclusion, fifth paragraph: ***The sensor array is also scalable. For example, one can extend the operation range by (a) increasing the number of sensors in the current line and (b) adding a parallel array to the current one. The enlarged array can be controlled with commercial fibre arrays, with up to 96 fibres and the same 127 μm spacing. This could extend the operation Y range in Fig. 5c by more than ten times to ~ 15 mm.***

3. In addition, the cw-ODMR requires a gold antenna to apply microwave field for NV centers. So, if the number of NV sensors is increased, the size of the antenna also increases. Please outline possible layout of the gold antenna if the number of NV sensors is increased.

We thank the Reviewer for raising this point. We have now identified two references (the new Ref. 43 and Ref. 44), showing homogenous microwave delivery over ~10 mm areas. We would also like to mention that dedicated site-defined microwave antennas, as demonstrated in our previous work (Ref. 22) can be used to drive a large array of spins power efficiently. We have included the discussion in the conclusion section of our manuscript.

Conclusion, fifth paragraph: ***Spin addressing over an extended area (at 10 mm scale) can be achieved with specially designed microwave antennas [22,43,44].***

4. The authors introduced the full 2D NV array could be used for sensing at the same time by replacing the 8-channel excitation fiber array with an eight-by-eight matrix fiber array (page 2). This is interesting, but how the each PL signal (8x8) can be separately detected through the eight waveguides?

We thank the Reviewer for the opportunity to clarify this idea. We believe this can be achieved by time-multiplexed readout (similar to what is shown in Ref. 12). To illustrate this in detail, we have now included a new supplementary section (Section J). Corresponding changes in the manuscript as follows:

Section- Scalable NV quantum sensing with photonic integrated circuits, last paragraph: ***Note that by replacing the 8-channel excitation fibre array with an eight-by-eight matrix fibre array, the full two-dimensional NV array could be used for sensing at the same time (i.e., for the same interrogation time) with spin readout multiplexed in time [12] (details can be found in Supplementary Section J).***

5. Fig. 1d shows the image of nanodiamond array. Although the PL of the selected eight nanodiamonds (by the yellow rectangle) show nearly uniform ODMR signal (Fig. 2b), I am wondering if other nanodiamond array, e.g., the right next line of the yellow rectangle, also show nearly uniform ODMR signal. Please comment if this is so.

We thank the Reviewer for the comment. We have now included a separate set of ODMR measurements on a different NV array where uniform contrast and resonance linewidth are again observed. The measurements are included in the new Supplementary Section B. Corresponding changes can be found in the main text.

Section- Parallel operation of eight NV magnetic sensors, second paragraph: ***We perform CW ODMR measurements of the eight sensors at the same time. With no external field (Fig. 2b), the ODMRs show resonances (fitted to Gaussian dips) with full width at half maximum (FWHM) ≤25 MHz and contrasts ($C = (I_{off} - I_{on})/I_{off}$, where $I_{off/on}$ are PL intensities off/on resonance) ~4% under a 31 dBm microwave driving power delivered at the source (the uniformity of the sensor spin resonance profile is supported by the results in Supplemental Section B).***

6. The spatial resolution of the photonic-integrated quantum sensor array is determined by the distance between the NV centers, which is ~125 μm (y-direction) in the present study and Ref. [15]. It seems that 100 nm diameter nanodiamonds can work in more tiny scales. Is it possible to make much smaller arrays to perform the sensing for nanorobot [Chem. Rev. Vol.121, 4999 (2021)] in the future?

We would like the take this opportunity to clarify. Firstly, unlike imaging the object directly through camera where the density of pixels in a given area (or effectively the pixel-to-pixel distance) decides the spatial resolution, the magnetic localisation technique, similar to the triangulation method used in Ref.7, has its *ultimate resolution* determined by the physical size of the sensor. That is, a change in the sensors' read is indistinguishable in any case when the object is moved by a distance smaller than the size of the sensor. The *practical resolution* is affected by other factors (such as the sensitivity and magnetic profile of the target) as summarised in Eq. 2. To clarify the differences, we have made the following changes to the manuscript.

Section-Static magnetic localisation through multi-point field reconstruction, Second paragraph: ***Through multi-point field reconstruction... Magnetic localisation, different from direct wide-field imaging [31,32], (1) does not require direct optical access to the target (since the target's field is recorded remotely by the sensors), and (2) has ultimate resolutions fundamentally limited only by the physical size of the sensors (similar to the localisation method used in [7])...***

Section- Static magnetic localisation through multi-point field reconstruction, last paragraph: ***The position estimation error, symbolising the practical magnetic-localisation resolution, is determined by factors beyond the physical size of the***

sensor (a few μm in our case), such as the sensor sensitivity and the local magnetic field gradient…

For tracking the nanorobot, we agree that it would be necessary to make the sensor array smaller to detect a larger enough field and field gradient. The current scheme would only be limited by the NV excitation, which with multi-core fibres (https://www.nature.com/articles/s41467-025-59037-1) can be downsized to sensor array ten time smaller of 10-20 μm, allowing the magnetic tracking of nanorobots with overall (nanowire) length of 5-10 μm (as shown in Chem. Rev. Vol.121, 4999 (2021)). We have now included the discussion in the new paragraph in the conclusion section.

Conclusion, fifth paragraph: ***In contrast, to track smaller magnetic robots (such as nanowire robots of overall length 5-10 μm [45]), sensor arrays with spacing ten times smaller (around 10-20 μm) can be achieved by bringing the parallel waveguides closer in the sensor region and optical excitation with multi-core fibres [46].***

7. In page 10, it is mentioned that microrobot's motion speed below 0.14 μm/sec is trackable using the photonic-integrated quantum sensor array. This means the microrobot can travel only ~0.5 mm/hour, which will be too slow to realize drug delivery. In the real applications, microrobot in blood tubes will move at a high speed of 600 μm/sec or higher than 1 mm/sec [e.g., Ref. (17) and Science Robotics vol.5, eaba5726 (2020)]. Is it possible to follow such a high-speed using photonic-integrated quantum sensor array with a frame rate of several mHz?

We thank the Reviewer for providing another reference on drug delivery. We clarify that the 600 μm/sec is out of the demonstrated regime with CW-ODMR on FNDs. However, this can be done with improved sensor sensitivity. See point 8 for detailed discussion.

We have carefully rewritten the manuscript to separate what has been experimentally demonstrated and what this photonic architecture could enable.

Conclusion, fourth paragraph: ***Looking forward, extending the demonstrated integrated architecture to substantially faster or rotation aware tracking will require major reductions in integration time through higher sensitivity and duty cycle (e.g., via improved spin resources and/or pulsed protocols), while accounting for practical overheads (i.e. thermal constraints). Maintaining $\delta_L \sim 50\ \mu m$ at F~10-20 Hz implies ~ 10^4 shorter integration than our current 360 s demonstrations; under Eq.2 this corresponds to ~10^2 improvement in effective sensitivity at fixed gradient. For context, nT/$\sqrt{Hz}$ level sensitivities have been demonstrated with NV ensembles under optimised conditions and protocols [28,41], indicating a plausible pathway to orders-of-magnitude improvements beyond the present on-chip nanodiamond CW-ODMR regime. Notably, the system-level advance demonstrated here--simultaneous, distinct, multi-channel readout of spatially separated NV sensors on a fibre-addressed photonic platform--provides a scalable hardware foundation (requiring photonic mode engineering similar to [42]) on which such sensing-protocol improvements can be deployed.***

We have also included the discussion for improved sensitivity, along with that for the next comment (comment point 8) in the manuscript, to consider both improved tracking speed and operation range.

8. Under the application to microrobot (the position tracking of a microrobot in Fig. 5), the estimation error increases much larger than 50 μm when the microrobot moves away from the good operation area (-750 μm). If the speed of 600 μm/sec or higher than 1 mm/sec is applicable, it will be difficult to track the motion of microrobot. Please discuss this issue?

We again thank the Reviewer for the comment. Following the discussion in point 7 and Eq.2 (*R=S/sqrt(t)/m*), the improved sensitivity can achieve a higher frame rate (smaller integration time *t*) and the detection of a smaller magnetic field gradient *m* with a fixed resolution. With sensor sensitivity improved to 1nT/sqrt(Hz) level (a 10^5 improvement as shown possible in Ref. 28 and Ref. 41), one can achieve tracking at 1.4 mm/sec (0.14 μm/sec*(10^2)^2) and the detection of a 10^-3 weaker field gradient. Following the power law decay of magnetic field (B ∝ 1/r^2, and dB/dr ∝ 1/r^3), this means that the operation range (with relation as shown in Fig. 5c) can be extended by 10 times to 0.75 mm*10=7.5 mm at least.

On top of this, similar to point 2, the operation range (the X range in Fig. 5b) can be further extended by adopting a second array parallel to the current one. This further doubles the 7.5 mm operation range mentioned above to 15 mm.

In conclusion, the 15 mm operation range and the 1.4mm/sec tracking speed can be achieved simultaneously with the improved sensor sensitivity. With these, we have now included the discussion in Supplemental Section I and the conclusion section.

Conclusion, fifth paragraph: ***The sensor array is also scalable. For example, one can extend the operation range by (a) increasing the number of sensors in the current line and (b) adding a parallel array to the current one. The enlarged array can be controlled with commercial fibre arrays, with up to 96 fibres and the same 127 μm spacing. This could extend the operation Y range in Fig. 5c by more than ten times to ~ 15 mm. A second parallel array, on the other hand, doubles the operation X range in Fig.5c. On top of that, by improving the sensor sensitivity to around 1 nT/$\sqrt{Hz}$ level, one can simultaneously increase the frame rate by 10^4 times and the operation range by another 10 times (see Supplementary Section I for detailed calculations).***

9. Overall, I feel this manuscript presents the new idea of photonic-integrated quantum sensor array based on cw-ODMR using NV centers in nanodiamonds. The current version is, however, not so appealing the broad range of readers since I am not sure if this photonic-integrated quantum sensor array can be used in real microrobot (and even nanorobot) applications. Probably, it will be possible to somehow improve the applicability of this array into biomedical system if the authors further discuss the layout of the photonic-integrated quantum sensor array.

We thank the Reviewer for appreciating the idea. Following point 6, 7, and 8, a new paragraph in the conclusion (included again below for completeness) now discusses the improved layouts and sensor sensitivity. We hope the proposed application in microrobot and nanorobot tracking is now convincing.

Conclusion, fifth paragraph: ***The sensor array is also scalable. For example, one can extend the operation range by (a) increasing the number of sensors in the current line and (b) adding a parallel array to the current one. The enlarged array can be***

controlled with commercial fibre arrays, with up to 96 fibres and the same 127 µm spacing. This could extend the operation Y range in Fig. 5c by more than ten times to ~ 15 mm. A second parallel array, on the other hand, doubles the operation X range in Fig.5c. On top of that, by improving the sensor sensitivity to around 1 nT/$\sqrt{Hz}$ level, one can simultaneously increase the frame rate by 10^4 times and the operation range by another 10 times (see Supplementary Section I for detailed calculations). Spin addressing over an extended area (at 10 mm scale) can be achieved with specially designed microwave antennas [22,43,44]. Notably, with the number of sensors significantly increased, machine learning based methods become the only way to efficiently extract the correlations between sensor readouts for magnetic inverse calculation. In contrast, to track smaller magnetic robots (such as nanowire robots of overall length 5-10 µm [45]), sensor arrays with spacing ten times smaller (around 10-20 µm) can be achieved by bringing the parallel waveguides closer in the sensor region and optical excitation with multi-core fibres [46].

We would also like to draw the Reviewer's attention to the new Supplementary Sections I and J, which discuss the magnetic tracking scalability and the full control and readout of a two-dimensional NV sensor array.

We thank all reviewers for their valuable comments which have significantly improved our manuscript, and for recognising our work. Please find attached a point-to-point response (in black) to the comments and suggestions raised by Reviewer 1 (in blue). **Corresponding changes (in red) are indicated in the *quoted paragraph (in Italic).*** With comments now thoroughly addressed we hope the work can now be published in Nature Communications.

The tip motion arrows in figure 4d-f gives the impression that the tip is moving continuously and in an arbitrary way. I don't see why the tip motion should be displayed any differently than it is shown as in Fig. 1a-c.

We thank Reviewer 1 for pointing this out. We have now made the presentation of tip movement consistent in Fig. 4 as suggested. Please see the updated Fig. 4d-f for changes.

Why not use higher order correlations to go beyond linear error scaling with the machine learning model? For N sensors and k subsets you would get ~N^k combinations (~N^2 for pairs, ~N^3 for triplets, etc.), which would lead to an error that scales as ~ N^(k/2). Is there some fundamentally unique property with pair information that disappears with larger group number in the machine learning model?

We thank the Reviewer for the opportunity to clarify. We agree that higher-order correlations can provide additional information if the features can be learned with a given machine-learning model complexity. In our experiment, we do not consider the contribution of higher-order correlations (n>=3) since they do not provide fundamentally unique information when the layout of sensors in one dimension is capturing a static magnetic field. Nevertheless, the higher-order correlations can be useful for capturing more complicated magnetic field profiles (which can be addressed following proposed method in Supplementary Section J). We have now included this clarification in the manuscript.

Section Static magnetic localisation through multi-point field reconstruction, fourth paragraph: ***…the number of pair-wise correlations (which scales with n^2) that can be extracted from n sensors, similar to the mathematical principle behind correlated and covariance sensing [7,9]. One should also note that higher-order correlations can, in principle, provide more information in magnetic localisation.***

Many claims in the conclusion section rely on dramatic improvements in sensitivity. While ~1 nT/sqrt(Hz) has been demonstrated in some NV ensembles (refs. 28, 43), they use different experimental platforms. The manuscript and SI assume this can be achieved. How could a sensitivity improvement by 100000x realistically happen for the experimental setup in the manuscript? Even driving with 100x laser power (at saturation) and increasing coherence times by 10x with pulse protocols cannot take ~100 uT/sqrt(Hz) to ~1 nT/sqrt(Hz).

The present sensors operate at approximately ~100 uT/sqrt(Hz) in the relevant field regime, whereas NV-ensemble platforms have achieved ~1 nT/sqrt(Hz) level sensitivities. To assess whether that scale is physically plausible in our photonic geometry, we provide a back-of-the-envelope estimate for replacing the present nanodiamond clusters with high-density CVD diamond. The estimated gain comes from narrower linewidth, larger participating NV number with corresponding improved photon flux, and pulsed magnetometry. The full discussion is included in the fifth section of the Supplementary Section I

Supplementary Section I, fifth paragraph*: In the main text, it is shown that the microrobot tracking frame rate can be improved significantly by improving the sensor sensitivity to around 1 nT/$\sqrt{Hz}$ [8,9]. This presents a 10^5 sensitivity improvement over the 100 μT/$\sqrt{Hz}$ shown by our current NV sensors. To see how 1 nT/$\sqrt{Hz}$ sensitivity can be explicitly achieved in our platform, we consider the integration of chemical vapour deposition (CVD)-grown diamond membranes (300 nm thick) on photonic waveguides. Commercial CVD diamond (DNV-B14, Element 6) has a high concentration of NVs (4.5 ppm of NVs) and T_2^* coherence time of 0.5 μs at room temperature [9]. The coherence results in ~50 times narrower linewidth $\Delta\nu = 1/(\pi\, T_2^*)$ than measured in this work. The increased NV concentration of this material implies 2.5×10^5 NVs at a positioned site of $4\ \mu m \times\ 0.4\ \mu m \times\ 0.3\ \mu m$). At saturation, this contributes ≈9 GHz count rate in our system (estimated by the saturated rate of a single NV in [7] and the improved collection efficiency presented in Fig. 1g of the main text). This presents an increased optical flux of 5×10^4 compared to the measured signal in our work. Following $\eta_{CW-ODMR} \propto \frac{\Delta\nu}{\sqrt{R}}$, with adopting improved NV linewidths and NV density, the CW-ODMR sensitivity can thus be improved by ~ 1000, and again by over ten times by considering pulsed ODMR sequences [8].*

Corresponding changes are also made in the Discussion Section:

Discussion, fifth paragraph: ***On top of that, by improving the sensor sensitivity to around 1 nT/$\sqrt{Hz}$ level** (which we show in Supplementary Section I is possible by leveraging high-density NV ensembles with μs-level T_2^* coherence time).*
